# High-throughput search of ternary chalcogenides for p-type transparent electrodes

**DOI:** 10.1038/srep43179

**Published:** 2017-03-07

**Authors:** Jingming Shi, Tiago F. T. Cerqueira, Wenwen Cui, Fernando Nogueira, Silvana Botti, Miguel A. L. Marques

**Affiliations:** 1 Institut Lumière Matière, UMR5306 Universitè Lyon 1-CNRS, Universitè de Lyon, F-69622 Villeurbanne Cedex, France; 2 Institut für Festkörpertheorie und -optik and ETSF, Friedrich-Schiller-Universität Jena, Max-Wien-Platz 1, 07743 Jena, Germany; 3 CFisUC, Department of Physics, University of Coimbra, 3004-516, Portugal; 4 Institut für Physik, Martin-Luther-Universität Halle-Wittenberg, D-06099 Halle, Germany

## Abstract

Delafossite crystals are fascinating ternary oxides that have demonstrated transparent conductivity and ambipolar doping. Here we use a high-throughput approach based on density functional theory to find delafossite and related layered phases of composition ABX_2_, where A and B are elements of the periodic table, and X is a chalcogen (O, S, Se, and Te). From the 15 624 compounds studied in the trigonal delafossite prototype structure, 285 are within 50 meV/atom from the convex hull of stability. These compounds are further investigated using global structural prediction methods to obtain their lowest-energy crystal structure. We find 79 systems not present in the materials project database that are thermodynamically stable and crystallize in the delafossite or in closely related structures. These novel phases are then characterized by calculating their band gaps and hole effective masses. This characterization unveils a large diversity of properties, ranging from normal metals, magnetic metals, and some candidate compounds for *p*-type transparent electrodes.

The delafossite mineral group is composed of ternary compounds with a general formula ABX_2_. This crystal structure, named after the French crystallographer Gabriel Delafosse, has been known since the 19th century, but it started to be intensively studied after the discovery in 1997 of *p*-type electrical conductivity in a transparent thin film of CuAlO_2_ delafossite^1^. Transparent conductive materials (TCMs) possess two usually mutually exclusive characteristics which are transparency and conductivity. TCMs are now essential components of many modern technologies as they are used as transparent electrodes for optoelectronic device applications^2^ (e.g., flat-panel displays and solar cells).

The atomic structure of delafossite crystals consists of planes of linearly coordinated A cations forming a triangular lattice, stacked between layers of flattened edge-sharing octahedra BO_6_. The cation A is usually a noble metal like Cu, Ag, Pd or Pt; the cation B is typically a transition or group 13 element or a rare earth species; finally, the anion X is usually oxygen, although few delafossites with other anions can be found in materials databases^3^,^4^,^5^. The trigonal delafossite structure (space group 166) is shown in the left panel of Fig. 1. A polytype of this structure with hexagonal symmetry (space group 194) can be formed when two neighboring A layers are stacked with each layer rotated by 180° in relation to one another. If A belongs to the IA group, one often finds another closely related trigonal structure (space group 164) with a slightly different stacking and where the layers are closer (see right panel of Fig. 1).

Due to the rather large range of possible stable configurations, it is not surprising that delafossites exhibit a significant richness in properties and potential applications. Several important technological applications have been proposed for members of this family, such as electrodes for hydrogen production by photo-electrochemical water splitting^6^,^7^, as luminescent materials^8^, in solar cells^9^,^10^,^11^, for thermoelectricity^12^,^13^,^14^,^15^, as possible transparent supercondutors^16^, etc. Recently, it was also found that delafossite PtCoO_2_ behaves like a nearly free-electron system, turning this surprising 5d transition-metal system in the most conductive oxide known^17^.

The discovery of *p*-type conductivity in CuAlO_2_ was particularly significant, as all technologically relevant transparent conducting oxides (TCOs), such as indium-tin-oxide (ITO) or Al- and Ga-doped ZnO (AZO and GZO), are *n*-type semiconductors. Unfortunately, neither CuAlO_2_ nor other recently proposed *p*-type TCOs possess adequate conductivity *and* transparency for technological applications.

The reasons for the difficulty of finding good *p*-type TCOs are well understood and they are related to the strong localization of holes at the valence band edge of most oxides. In fact, the O-2*p* orbitals, which form hole transport paths at the top of the valence band of many oxides, are rather localized and generally much lower in energy than the valence orbitals of the metal species. These two facts imply difficulties in hole doping and large hole effective masses (and therefore low hole mobilities).

In the race for obtaining such elusive material, delafossites have been widely explored for the past years. Getting inspiration from the structure/property relations in the delafossite family, Hosono and co-workers^18^,^19^ proposed few empirical rules for *p*-type TCOs, aiming at favoring at the same time large band gaps and dispersive top valence bands: (i) The A cations should possess closed *d* shells to prevent from absorbing light in the visible range. Optical transparency has also been associated with dipole forbidden transitions related to the linear O-A-O coordination motif. (ii) The energy levels of the A cations should overlap with the anion *p* levels (O 2*p*) to allow for strong hybridization at the top of the valence band. (iii) Hole paths should be favored, for example providing B states that can mix strongly with O 2*p* states into the valence band to build a A-O-B-O-A pathway.

Following Hosono’s rules, the discovery of *p*-type conductivity in CuAlO_2_ triggered the experimental and theoretical characterization of other delafossites^20^,^21^,^22^,^23^,^24^ and of transparent *p*-*n* junctions. CuCrO_2_ and CuGaO_2_, are also *p*-type TCOs^25^,^26^. CuInO_2_^27^ is particularly interesting because it is amphoteric, i.e. it can be alternatively acceptor-doped or donor-doped. This ability is explained by the unique presence of two cationic environments in the delafossite structure: an octahedral indium environment that favors strong hybridization and *n*-type dopability, together with a linear copper environment that favors hybridization at the top valence and *p*-type doping^24^. Among delafossite compounds, CuCr_1−*x*_Mg_*x*_O_2_^28^ possesses the highest conductivity of 220 S cm^−1^, but transparency in the visible range is only about 30%, much smaller than for the traditional *n*-type TCOs. Recently synthesized Cu deficient CuCrO_2_ has a conductivity in the range of 1–12 S cm^−1 ^29. It was also claimed that the transparency can be improved by increasing the temperature, at the cost of higher resistivity^28^,^30^,^31^. Recently, Barnabé and co-workers studied Mg-doped CuCrO_2_ thin films by using RF-sputtering^32^. They found that at 600 °C, the Mg-doped CuCrO_2_ structure displayed an optical transmittance of only 63% in the visible range with, however, a conductivity of 1.6 S cm^−1^.

Another idea, again put forward by Hosono and co-workers^33^, is to replace oxygen with a chalcogen (S, Se, or Te) with more delocalized *p* orbitals. This has led to the synthesis of LaCuOS and LaCuOSe *p*-type oxides^34^,^35^. Unfortunately, these latter compounds have either a too low mobility (LaCuOS) or a too small band gap (LaCuOSe). However, this strategy looks appealing as the *p* states of the chalcogen atoms are usually higher and therefore make the compound more prone to be *p*-doped. Recent calculations suggest that this is the case for Ba(Cu, Ag)_2_(S, Se)_2_ compounds^36^.

Going beyond simple empirical rules, experimental databases have been recently explored by performing high-throughput *ab initio* calculations^37^,^38^ to single out compounds with large band gaps (>3 eV) and low effective masses. These previous investigations focused on oxides and showed that small hole effective masses are much rarer that small electron effective masses. Hautier *et al*.^37^ found that hole effective masses of 0.5–1.0 electron masses (*m*_*e*_) in transparent oxides like PbTiO_3_, PbZrO_3_, PbHfO_3_, K_2_, and Tl_4_V_2_O_7_ are due to the hybridization of the oxygen 2*p* orbitals with *s* states of Sn^2+^, Pb^2+^, Tl^1+^. Similarly, hole effective masses smaller than 1.0 *m*_*e*_ and band gaps larger than 3.0 eV are reported by Sarmadian *et al*.^38^ for X_2_SeO_2_, with X = La, Pr, Nd, and Gd. Recently, Cerqueira *et al*.^39^ performed a search of new compounds of the form (Cu, Ag, Au)XO_2_ by combining global structural prediction methods and high-throughput calculations. They showed that there are 45 new (quasi) stable phases in this system, some of which are candidate *p*-type transparent conductors. However, these new stable phases contain Ag and Au, which are not ideal elements for commercial applications.

We remind that finding a large band gap and low hole effective masses is not a sufficient conditions for a good *p*-type TCO. In fact, another essential condition, much harder to translate to the minimization/maximization of a simple material property, is the *p*-type dopability of the system. As an example, we can think about ZnO: the hole effective mass values for ZnO range from 0.31 *m*_*e*_ to 0.59 *m*_*e*_ and its band gap is about 3.5 eV. Despite these ideal parameters, numerous attempts to produce *p*-type doped ZnO have failed^24^.

It appears by now clear that if we work only with known materials it will be very hard to improve both transparency and conductivity to technologically acceptable limits. However, the search for improved *p*-type TCMs remains an important open question. Fortunately, we only know experimentally a fraction of all possible stable compounds. Delafossite structures, which combine the advantages of linear and octahedral environments for the two cations remain particularly attractive for *p*-type TCMs and ambipolar doping. We therefore asked ourselves: Are there more delafossite compounds that wait to be synthesized and characterized? And more precisely: can good *p*-type TCMs be found in the delafossite family or should we resign ourselves and direct future search towards completely different crystal families?

In this Article we provide an answer to the questions above performing a comprehensive first-principles investigation of ABX_2_ systems that may crystallize in the delafossite structure. We extend in fact our study to all ABX_2_ compositions where A and B cover the periodic table from H to Bi (excluding the rare gases and the lanthanides) and X is a chalcogen (O, S, Se, and Te). This set contains 63 × 62 × 4 = 15624 compositions and includes the already known delafossites crystals. At this point, we take all A and B elements and do not make any bias towards non-toxic or abundant elements. Such filtering can however be easily performed *a posteriori*.

We note that most research on delafossites is restricted to oxide materials. Also most of the systems known have X = O. For example, the materials project database^3^, contains 90 trigonal ABX_2_ oxides (with space group 166) while only 23 sulfides, 10 selenides and 7 tellurides. It is unclear if this is because the delafossite structure prefers the highly electronegative oxygen, or if it simply reflects a bias against sulfides, selenides, and tellurides.

To screen the large amount of possible compositions of ABX_2_ compounds, we use a combination of high-throughput density-functional theory^40^ and global structural prediction methods^41^. This two-step process is essential to ensure that our predicted structures are (i) thermodynamically stable, i.e. that do not decompose to other more stable species, and (ii) are indeed delafossites, i.e. that there are no other lower energy phases for the given composition. The resulting stable delafossite phases are then characterized theoretically using state-of-the-art *ab initio* methods. The final results provide precious indications for further experimental synthesis and open the way for more specific theoretical and experimental studies of dopability and transport properties, which can be restricted only to the most interesting candidates.

## Results

### Overview

The main results of our high-throughput search are summarized in the stability maps of Fig. 2, where we plot the distance to the convex hull of the delafossite phase for all the compositions studied here. In order to put into evidence the chemical similarity between the chemical elements, we order them using an optimized Pettifor scale^42^,^43^. Each point in the plots corresponds to a different compound, and the color code is used to indicate the distance to the convex hull of stability (light green – stable, red – unstable). It is quite evident that the large majority of the compositions are highly unstable in the delafossite phase, with distances to the hull larger than 400 meV/atom. However, there are still many systems that are either thermodynamically stable or close to stability.

We then filter this list using a threshold of 50 meV/atom above the convex hull. This is a rather conservative threshold, that tries to minimize the number of false positives, but at the same time takes into account the errors in evaluating differences of PBE energies^44^ and the possible reduction of the energy of formation related to small distortions of the lattice, native defects, temperature and disorder, etc. This also allows us to include in our list some experimentally known delafossite structures that exhibit (small) positive distances to the hull in our formalism, such as CuInO_2_ (7 meV/atom).

Within this threshold we find 285 compositions (109 oxides, 66 sulfides, 67 selenides, and 43 tellurides). We can find entries in the materials project database for 101 of the 285 compositions, namely for 72 oxides (including our predictions of ref. 39), 11 sulfides, 11 selenides, and 7 tellurides. Obviously, there are many more entries ABX_2_ with X = O, S, Se, Te in the materials project database (463 in total at the time of writing), but most of these, such as, e.g., the chalcopyrites, have atomic arrangements very different from the delafossites, and do not appear therefore as a result of our search. We could also find in literature some (unfortunatley incomplete) information on other compounds that are not part of the materials project database: MgNiO_2_, PdCrO_2_^45^,^46^, PdRhO_2_^46^, KBiS_2_^47^, KScS_2_^48^, KYS_2_^48^, RbScS_2_^48^, AgRhS_2_^49^, AgYS_2_^50^, HgTiS_2_^51^, TlMnS_2_^52^, TlMnSe_2_^52^, KCrSe_2_^53^, CsBiTe_2_^54^, TlYTe_2_^55^. The fact that we also identify these compounds as thermodynamically stable is another confirmation of the validity of our approach.

It is quite interesting to compare at this stage these numbers. First, they indicate that there are probably many more stable ternary compounds than the ones known experimentally (or predicted theoretically and already present in the databases). Second, we can see the strong experimental bias towards oxides in detriment of other chalcogenides.

Up to now, we only know that the delafossite phase is stable (or almost) regarding the decomposition into other compounds. The following step in our investigation is to evaluate if the delafossite phase is indeed the ground-state structure, or if there exist other phases lower in energy. We combined two different approaches for all stoichiometries that did not already appear in the materials project database: (i) We tested a set of common prototype structures for the composition ABX_2_ (see Table 1). This amounts to 28 further structural optimizations per composition. (ii) For each composition, we performed two independent structural prediction runs including 8 atoms (2 formula units) in the unit cell. The length of each run was tuned in order to obtain ~50 minima.

Our final step consists in filtering again the results and characterizing the remaining materials. We retain only the stoichiometries that are thermodynamically stable or slightly unstable (up to 25 meV/atom above the convex hull, which corresponds approximately to room temperature), and whose ground state structure is a delafossite or a closely related structure. We define as closely related structure a crystal structure that maintains the essential characteristics of the delafossite: the presence of both a linear and an octahedral environment for the two cations. Note however, that the linear and octahedral coordinations can be significantly distorted in some quasi-delafossites. For these structures we finally calculate indirect and direct band gaps, magnetic moments per formula unit and hole effective masses whenever the system is non-metallic. Our results concerning all new structures, i.e. the ones *not already present* in the materials project database, are summarized in Tables 2, 3, 4, and 5. For completeness, we decided to also include the information concerning the phases reported in the literature but not present in the materials project database. These can be found in bold in the tables. All crystallographic information files are given as Supporting Information.

We can observe that there are 79 delafossite systems that are stable (and 123 close to stability). The tables include 16 (10) new oxides, 40 (27) sulfides, 38 (25) selenides, and 29 (17) tellurides. (The numbers in parentheses are the number of phases strictly below the hull of stability). The list includes a series of rather exotic systems that depart from the usual A^+^B^+3^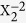 oxidation states of the delafossites. Actually, we do find often an element of the IA group (Li, Na, K, Rb, or Cs) or IB group (Cu, Ag, Au) in position A. However many other possibilities appear such as Hg, In, Tl, Bi, or even halogens such as Br or I.

**Figure 1:**
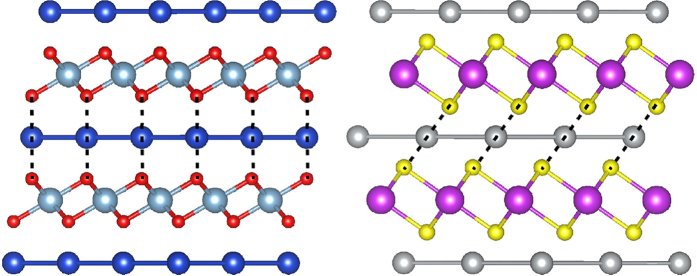
Left: Trigonal delafossite structure (space group 166) of CuAlO_2_ (Cu dark blue, Al cyan, O red); Right: Trigonal structure (space group 164) of AgBiS_2_ (Ag gray, Bi magenta, S yellow). From this perspective, there is no difference between this phase of AgBiS_2_ and the trigonal (space group 166) structure of TlScSe_2_. The A cations of ABX_2_ form a triangular lattice that alternates with layers composed of distorted edge-sharing BX_6_ octahedra. The similarity between the left and right panels is evident: only the alignment of A and X atoms differentiates the two structures (the dotted lines are a guide to the eye meant to emphasize this difference).

**Figure 2:**
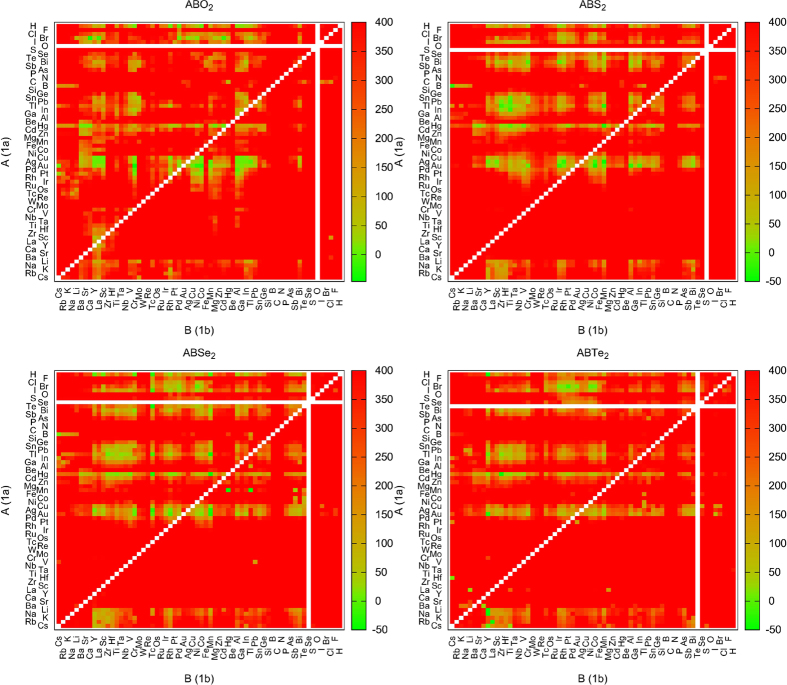
Stability maps for ABO_2_, ABS_2_, ABSe_2_ and ABTe_2_. The colors indicate the distance to the convex hull of stability (in meV per atom), with green meaning that the composition is thermodynamically stable. The order of the atoms along both axes follows an optimized Pettifor scale.

Most of the new stable compositions crystallize either in the trigonal delafossite structure of, e.g., CuAlO_2_, or in the closely related trigonal structures (see Fig. 1) of, e.g., AgBiS_2_ (space group 164) or TlScSe_2_ (space group 166). However, we also find a variety of structures showing small distortions or different stacking patterns in comparison to the standard delafossite arrangement. We will now discuss in detail our results separately for oxides, sulfides, selenides, and tellurides.

### ABO_2_

Among the 3906 possible delafossite oxides (compatible with our selection of chemical elements) there are 93 phases that appear at least 25 meV/atom below the convex hull of stability. Of these, 77 can already be found in the materials project database^3^ or in ref. 39. The remaining 16 compositions are reported in Table 2.

All compounds with A = Pd, Pt, Hg, Tl, and Br crystallize in the delafossite structure with space group 166. The only exception is BrLaO_2_ where the stacking is slightly changed, leading to a monoclinic ground-state (prototype of NaCuO_2_) which is 14 meV/atom lower in energy than the trigonal delafossite phase. On the other hand, when A is an alkali or an alkali earth metal, the ground-state phase has the trigonal structure of TlScSe_2_ (space group 166), that is very similar to the trigonal structure of AgBiS_2_ (space group 164) shown in the right panel of Fig. 1.

The crystals KInO_2_, RbInO_2_, RbRhO_2_, CsLaO_2_, HgMgO_2_, and BrLaO_2_ are semiconductors with gaps between 3 and 4 eV, and not too large effective masses of around 2 *m*_*e*_ (with the notable exception of RbRhO_2_). We note that hole effective masses of 1~2 *m*_*e*_ are not small in absolute terms, but they are comparable with those of CuAlO_2_ and CuCrO_2_. Among the metals, the compounds containing Ni and Cr are magnetic, with a total magnetization that can reach 3.0 bohr magnetons for the ferromagnetic compound PdCrO_2_.

### ABS_2_

Among all the stable sulfides that we found in this work, only 10 compositions could be found in the materials project database^3^. These are NaScS_2_, NaInS_2_, KCrS_2_, KLaS_2_, RbYS_2_, CsLaS_2_, AgBiS_2_, AuCrS_2_, TlBS_2_, and TlInS_2_. All the other novel 40 compositions can be found in Table 3. There are 6 systems with A = Ag and 9 with A = Au, however we did not find any new system with A = Cu. Most of these have the standard delafossite structure with the exception of AgScS_2_ and AgYS_2_ that exhibit a different stacking of the layers (space group 160 and 156 respectively). We also find 5 systems with A = Hg, 4 with A = Bi, 4 with A = Tl, and 3 with A = Pb. Interestingly, we also find a couple of delafossites with trivalent In and tetravalent Sn in the Wyckoff 1a position. Finally, there are a series of systems with an alkali in the 1a position, all assuming the trigonal lattice depicted in Fig. 1. The only systems that do not crystallize in one of the structures of Fig. 1 are HIrS_2_ and HgTiS_2_. While the former just exhibits a different stacking, in the latter Hg forms layers with square and not hexagonal symmetry.

Many of the novel systems are semiconducting with indirect band gaps varying in the rather large range of 0.7–3.4 eV. Hole effective masses, as expected considering the more delocalized nature of S *p* states, are significantly lower than in oxides. We would like to point out materials like (K, Rb)YS_2_, composed of relatively abundant elements, with rather large band gaps of the same order of magnitude as the usual Cu delafossite TCOs, and with rather small hole effective masses (of the order of 1.2–1.3 *m*_*e*_), if compared to CuAlO_2_. AgYS_2_, with an HSE band gap of 3.16 eV and a hole effective mass of 0.71 *m*_*e*_, is the best system identified in this study. Again, stable metals with Cr or Mn in the 1b position are magnetic with relatively large magnetization.

### ABSe_2_

For the selenides ABSe_2_ we find 48 phases below 25 meV/atom, 10 of which can be found in the materials project database. The results for the remaining 38 phases can be found in Table 4. For the compounds with a IA element in the 1a Wyckoff position we find that stable compounds tend to have larger alkalis (Rb, Cs) in detriment of the lighter ones (Na, K). The same does not seem to happen with the IB group, and we can observe several compounds containing S and Se together with both Ag and Au. We also witness the appearance of the rather exotic INiSe_2_ at the limit of our threshold of 25 meV/atom.

Again most of the compounds crystallize in one of the structures of Fig. 1, with the following exceptions: HScSe_2_ has a monoclinic ground-state structure where the layers of H are buckled; HgTiSe_2_, like other Hg compounds, has Hg layers with square symmetry; In HgVSe_2_ we find a different stacking of the layers leading to a monoclinic ground-state; HgNbSe_2_ and TlVSe_2_ present BSe_2_ layers that are distorted with respect to the usual delafossite structure; Finally, SbMnSe_2_ has buckled Sb planes.

We again obtain the usual mixture of metals and semiconductors, although, and as expected, with gaps generally smaller than for the sulfides. Compounds including Cr and Mn are magnetic, but AuCoSe_2_, BrNiSe_2_, and INiSe_2_ are non-magnetic.

### ABTe_2_

From the systems found in our search, there are only 7 ABTe_2_ phases in the material project database. These structures are AgSbTe_2_, AgBiTe_2_, KLaTe_2_, KYTe_2_, TlSbTe_2_, TlScTe_2_ and TlBiTe_2_. Besides these known tellurides, we identify 29 new phases that are stable or very close to stability (see Table 5). As expected by the chemical similarity between Se and Te, the situation here is analogous to what was described for the Se systems. Perhaps the biggest difference regards the chalcogen in the Wyckoff position 1a. We find a series of Br and I metallic compounds with the 1b position occupied by a transition metal. Some of these systems, like BrPtTe_2_, are quite below the convex hull of stability, which, together with the overall consistency of the results, makes us believe that these exotic systems can be experimentally synthesized.

From the compositions that do not crystallize in the structures of Fig. 1 we have: HgVTe_2_, with displays distorted planes; BrIrTe_2_ with has a different stacking; BrPdTe_2_ with distorted Br layers; finally INiTe_2_ and IPdTe_2_ have a different stacking.

The two compounds with Mn in the 1b position are ferromagnetic, but, more surprisingly, we also find that HgVTe_2_ are TlTiTe_2_ have a relatively small magnetic moment. For the semiconducting compounds there is also a further decrease of the magnitude of the gap with respect to the selenides.

### Gaps and hole effective masses

**Figure 3:**
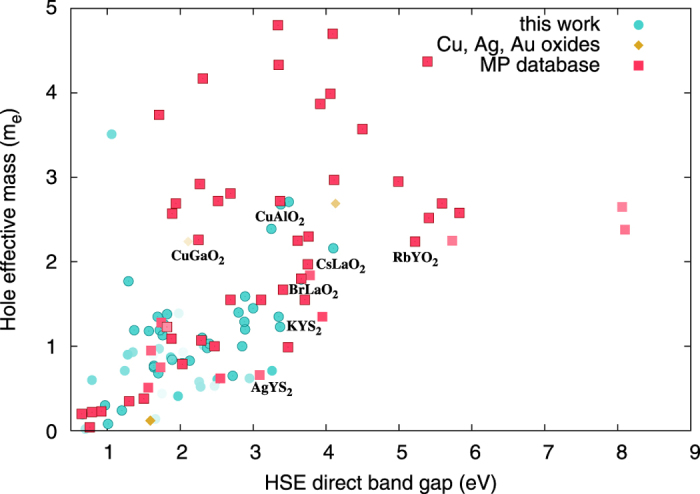
Hole effective masses as a function of the HSE06 band gap for all compounds that are thermodynamically stable.

Finally, Fig. 3 is a scatter plot of the direct band gap and the average hole effective mass for all delafossite-like compounds within our window of stability. We include both the new systems found in this work, as well as the other compounds from the materials project database (such as CuAlO_2_, CuGaO_2_, RbYO_2_, AgYO_2_, etc.), and the Cu, Ag, and Au oxides from ref. 39. Such a plot allows us to quickly recognize potential outliers for *p*-type transparent conductors. In particular, we are interested in compounds in the lower central part of the plot, with a band-gap larger than the visible range and a small hole mass. We chose to plot the direct band gap, as this is the one most directly related to optical absorption. The numbers were calculated with the HSE06^56^ approximation to the xc potential of DFT, that usually gives accurate and systematic gaps. We should keep in mind, however, that HSE underestimate systematically the band gap of medium-large gap semiconductors. Moreover, some of these materials can have substantial quasi-particle corrections and large excitonic binding-energies^57^,^58^. The hole masses discussed here, on the other hand, are averaged values, and may not include all the physics in cases of valence bands with multiple valleys.

We also label a few relevant points in Fig. 3, such as CuAlO_2_ and CuGaO_2_. It is evident, for example, that CuAlO_2_, synthesized for the first time on 1997^1^, possesses a large band gap of around 3.37 eV (in good agreement with the experimental value of 3.53 eV^59^), but has a relatively high hole effective mass (≈ 2.5). We can observe several new phases that possess a sufficiently large band gap and a smaller hole effective mass. These phases are not only oxides, but also sulfides, and few selenides and telluride. For example, some candidate *p*-type TCO systems are BrLaO_2_ (gap 3.92 eV, hole mass 2.23), CsLaO_2_ (gap 4.11 eV, hole mass 2.16), KYS_2_ (gap 3.37 eV, hole mass 1.23), AgYS_2_ (gap 3.16 eV, hole mass 0.71).

Looking at the branch point energies of these candidates, we found a similar behavior for all of them, with values that lie in the middle of the HSE band gap, at distances of 1.4–1.9 eV from the lowest conduction band. The fact that the branch point energy is not close to or inside the conduction band is good news, as that would imply that *p*-type doping is very difficult (as in the case of ZnO). However, this result does not allow to conclude that it is possible to *p*-dope these materials. When the branch point energy is in the middle of the gap all scenarios are in fact open: the compound may be *p*- or *n*-type dopable, or even ambipolar. Accurate defect calculations are then the only safe way to draw reliable conclusions.

## Conclusions

In this article, we performed a high-throughput investigation using density functional theory and combining calculations of crystal prototypes with structural prediction. We focused on ternary compounds of composition ABX_2_, where A and B are elements of the periodic table up to Bi, and X is a chalcogen (O, S, Se, and Te) in search of thermodynamically stable delafossite or closely related structures. The starting set of compositions was made of 15 624 compounds, for which we found the lowest-energy structure under the constraint to preserve the symmetries of the trigonal delafossite structure. After this step 285 compounds turn out to be within 50 meV/atom from the convex hull of stability. These compounds were further studied using the minima hopping method for global structural prediction, removing any symmetry constraint to obtain the ground-state crystal structure. At the end of this procedure, we could count 79 ternary systems not present in the materials project database that are thermodynamically stable (and therefore potentially synthesizable) and crystallize in the delafossite or in closely related structures. Among these new delafossites, 10 are oxides, 27 sulfides, 25 selenides and 17 tellurides. If we include also the new structures that are above the convex hull of stability within a threshold of 25 meV per atom, the number of new systems increases to 123.

By calculating the band gaps and hole effective masses of these structures we identified few systems that could be better *p*-type transparent conducting materials than those already known. In particular, AgYS_2_, with an HSE band gap of 3.16 eV and a hole effective mass of 0.71 *m*_*e*_, is probably the best system that emerged from this study. KYS_2_ and RbYS_2_ have even larger band gaps (more than 4 eV) and effective masses of only about 1 *m*_*e*_. We also found several systems, mainly containing Cr and Mn, that have relatively large magnetization densities.

These results provide us with a much more complete picture of the delafossite structure and can be used as a guide for further experimental and theoretical exploration of this fascinating family of compounds. The crystal structures of these new compounds are now available (see Supporting Information and ref. 3) for more accurate theoretical characterization, hoping that some other interesting properties that have not been screened in this first study can come on the scene and motivate experimentalists to try to synthesize some of these compounds.

## Methods

As starting prototype unit cell we used the rhombohedral (space group 166) delafossite structure, and not the hexagonal (space group 194) one. In fact, these two layered phases only differ in the stacking of the layers, and we have verified^39^ that they exhibit very similar total energies (with differences of the order of few meV/atom) and electronic properties. In that sense these two crystal structures are equivalent in our calculations. However, the former contains 4 atoms in the primitive unit cell, while the latter contains 8, which would lead to a considerable increase of the computational burden of our high-throughput search.

As the A and B atoms are in the inequivalent crystallographic positions 1*a* and 1*b* of the delafossite structure, there are 15 624 possible ABX_2_ compositions that combine the 64 chemical elements considered here. It is true that this number could be quite reduced by eliminating compositions on the basis of empirical rules based on oxidation states or the radius of the A, B, and X elements. However, we decided not to follow this path in order to allow for delafossites that do not conform to our intuition.

For each one of these structures we optimized the geometry and calculated the total energy. This was done within ab initio density functional theory as implemented in the computer code VASP^60^,^61^. All parameters were set to guarantee compatibility with the data available in the materials project database^3^ and open quantum materials database^4^. We used the PAW^62^ datasets of version 5.2 with a cutoff of 520 eV and Γ-centered *k*-point grids, as dense as required to ensure an accuracy of 2 meV/atom in the total energy. All forces were converged to better than 0.005 eV/Å. We followed the same protocol as in ref. 39: spin-polarized calculation using the Perdew-Burke-Ernzerhof 63 (PBE) exchange-correlation functional, with the exception of oxides and fluorides containing Co, Cr, Fe, Mn, Mo, Ni, V, W, where an on-site Coulomb repulsive interaction U with a value of 3.32, 3.7, 5.3, 3.9, 4,38, 6.2, 3.25, and 6.2 eV, respectively, was added to correct the *d*-states. We are of course aware that the PBE (+U) functional is not always able to determine the correct electronic and spin ground-state. Moreover, it is well-known that the PBE, as well as many other exchange correlation functionals, including hybrid functionals^64^, yield an average error for the energies of formation substantially larger than the so-called chemical accuracy (around 1 kcal/mol ≃ 43 meV)^44^. Nevertheless, the optimized crystal structure and the ordering of low-energy phases, resulting from differences of formation energies, is very often qualitatively and quantitatively good within PBE (+U), even in many cases in which the electronic states are not correctly described.

The second step of our search is then to identify which compositions are thermodynamically stable. A composition is thermodynamically stable if it does not decompose (in a possibly infinite time) to other phases. In other words if its free energy is lower than the free energy of all decomposition channels, not only in elementary substances but also on other possible binary, ternary, etc. (reservoir) compounds. In this case we say that the structure lies on the convex hull of stability. We worked always at zero temperature and pressure, and we neglected the effects of the zero point motion of the phonons (that are expected to be negligible for these materials). In this case, the thermodynamic quantity of interest is the total energy. Fortunately, it is no longer necessary to calculate the total energy of all possible reservoir compounds, as this information is already available in excellent public databases, such as the materials project^3^, open quantum materials database^4^, and the ab-initio electronic structure library aflowlib^5^. We chose to use the materials project database for our reference energies, and to determine the distances to the convex hull of stability with pymatgen^65^. The materials project database includes most of the experimentally known inorganic crystals that are present in the ICSD database^66^,^67^ and an increasing number of theoretically predicted phases, including those proposed in ref. 39. Note that, as we only use the compounds included in the database to build the convex hull, new materials will appear with negative distances to the hull.

The next step is to perform global structural prediction runs for the most stable structures stemming from the high-throughput runs. There are several good algorithms available in the market for this task, such as genetic algorithms^68^, particle swarm methods^69^,^70^, random search^71^, etc. Our method of choice is the minima hopping method^72^,^73^. This is an efficient crystal-structure prediction algorithm designed to determine the low-energy structures of a system given solely its chemical composition. At a given chemical composition the adiabatic potential-energy surface is explored by performing consecutive short molecular dynamics escape steps followed by local geometry relaxations taking into account both atomic and cell variables. The initial velocities for the molecular-dynamics trajectories are chosen approximately along soft-mode directions, thus allowing efficient escapes from local minima and aiming towards low-energy structures. Revisiting already known structures is avoided by a feedback mechanism. The minima hopping method has already been used for structural prediction in a wide range of materials^39^,^74^,^75^,^76^,^77^,^78^, including the dependence on pressure and temperature, with remarkable results.

Having in mind the possible final application as TCM, for a first characterization of the structures we determined hole effective masses and band gaps. For the calculation of effective masses we used the same framework as for the calculation of the total energies, that relies on the PBE functional. Effective masses were calculated with the program BoltzTrap^79^ for a concentration of 10^18^ cm^−3^ and a temperature of 300 K. We remark that while it is clearly desirable to have dispersive valence bands close to the band gap to ensure high hole mobilities, this is not the only possibility as other transport mechanisms, e.g., based on small polarons, have been proposed for delafossite *p*-type TCOs^24^.

Although the PBE functional is a quite reasonable approximation for the curvature of the bands, it is well known that this functional underestimates considerably the band gaps. Therefore we decided to adopt the hybrid exchange-correlation functional of Heyd, Scuseria, and Ernzerhof (HSE06)^56^ to obtain reliable band gaps.

Finally, we give a first estimate of the *p*-type dopability by testing if acceptor-like defects can be easily formed in the system and whether one can expect or not the generation of compensating native defects (e.g., anion vacancies). To this purpose, a simple criterion with significant predictive power is the determination of the branch point energy. The branch point energy^38^,^80^,^81^, or charge neutrality level, is defined as the energy at which the defect states induced in the gap change their character from predominantly donor-like to acceptor-like. The position of this energy with respect to the band edges is particularly important as the formation of acceptor (donor) defect states in the gap becomes favorable above (below) it. Hence, in a material with a branch point energy lying in the conduction band (such as ZnO) or high in the band gap, donor impurity defects tend to be shallow, while acceptor impurities, if they can exist, will be necessarily deep. The condition to favor *p*-type doping is therefore to have the branch point energy at least in the middle of the gap. In general, the lower the value of this energy the easier *p*-type doping can be expected. To calculate this quantity we follow Schleife *et al*.^80^ and we define it as a weighted average of the midgap energies (calculated with a hybrid HSE06 functional) over the Brillouin zone. The number of valence and conduction states included in the calculation follows the presciption of ref. 80, which has already been adopted in other works^38^,^81^. This method is a particularly convenient criterion to use in high-throughput calculations as it only requires the energy levels of the perfect crystals. We note, however, that reliable conclusions on the *p*-type dopability of the system can be obtained only by performing extensive defect calculations.

Crystal structures were visualized with VESTA^82^.

## Additional Information

**How to cite this article:** Shi, J. *et al*. High-throughput search of ternary chalcogenides for p-type transparent electrodes. *Sci. Rep.* **7**, 43179; doi: 10.1038/srep43179 (2017).

**Publisher's note:** Springer Nature remains neutral with regard to jurisdictional claims in published maps and institutional affiliations.

## Supplementary Material

Supplementary Dataset 1

Supplementary Information

## Figures and Tables

**Table 1:** Common prototype structures for the composition ABX_2_ used in this study.

spg	^#^atoms	example
166	4	CuAlO_2_
122	8	CuInSe_2_
62	16	CaBaO_2_
14	16	CuBrTe_2_
15	8	KFeSe_2_
141	8	CuGaSe_2_
12	4	NaCuO_2_
63	8	CuSrO_2_
15	32	KAlTe_2_
140	8	KAlTe_2_
160	4	CuAsSe_2_
33	16	LiAlSe_2_
123	4	AgSbTe_2_
9	8	AgInSe_2_
156	4	AgAlS_2_

We list the space group (spg) number, the number of atoms in the unit cell, and an example of a composition that crystallizes in the phase.

**Table 2:** Distance to the convex hull (*E*_*hull*_ in meV/atom), HSE06 gaps (indirect and direct in eV), average hole effective mass for the semiconducting phases ( 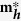 in *m*_*e*_), magnetic moment per formula unit (*μ* in bohr magnetons, and space group (spg) of the new (quasi) stable delafossite and closely related phases with composition ABO_2_.

	*E*_hull_	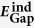	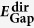	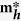	*μ*	spg
KInO_2_	−106	3.49	3.63	2.71	0	166^b^
RbInO_2_	−27	3.38	3.57	2.68	0	166^b^
RbRhO_2_	−185	3.06	3.49	34.84	0	166^b^
CsLaO_2_	−44	4.10	4.15	2.16	0	166^b^
**MgNiO**_2_	−4	0	0	—	2.0	166^b^
PdAlO_2_	−7	0	0	—	0	166^a^
**PdCrO**_2_	−12	0	0	—	3.0	166^a^
**PdRhO**_2_	22	0	0	—	0	166^a^
PtAlO_2_	12	0	0	—	0	166^a^
PtNiO_2_	17	0	0	—	1.0	166^a^
HgMgO_2_	0	3.25	3.77	2.39	0	166^a^
TlRhO_2_	23	0	0	—	0	166^a^
BrCdO_2_	−29	0	0	—	0	166^a^
BrLaO_2_	−13	3.92	4.59	2.23	0	12^a^
BrNiO_2_	14	0	0	—	1.4	166^a^
BrTlO_2_	24	0	0	—	0	166^a^

The compounds that are not in the materials project database, but that are reported in literature are indicated in bold. ^a^See left panel of Fig. 1. ^b^See right panel of Fig. 1.

**Table 3:** Properties of delafossite sulfides ABS_2_.

	*E*_hull_	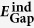	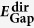	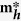	*μ*	spg
HIrS_2_	0	2.49	2.81	1.13	0	160
**KBiS**_2_	−184	2.14	2.53	5.09	0	166^b^
**KScS**_2_	−158	2.89	3.72	1.20	0	166^b^
**KYS**_2_	−194	3.37	4.14	1.23	0	166^b^
**RbScS**_2_	−137	2.88	3.69	1.29	0	166^b^
AgCoS_2_	−22	1.29	2.50	1.77	0	166^a^
AgMnS_2_	−53	0	0	—	2.1	166^a^
**AgRhS**_2_	−26	1.69	2.19	1.35	0	166^a^
AgScS_2_	−3	2.29	3.06	0.66	0	160
AgIrS_2_	11	1.87	2.16	0.87	0	166^a^
**AgYS**_2_	−15	3.16	3.24	0.71	0	156
AuAlS_2_	−29	2.72	2.97	0.65	0	166^a^
AuBiS_2_	20	1.35	2.28	0.93	0	166^a^
AuCoS_2_	−48	1.36	2.68	1.19	0	166^a^
AuInS_2_	−13	1.70	1.95	0.68	0	166^a^
AuIrS_2_	−23	2.13	2.52	0.83	0	166^a^
AuMnS_2_	−41	0	0	—	2.0	166^a^
AuRhS_2_	−54	1.72	2.39	1.19	0	166^a^
AuScS_2_	24	2.28	3.14	0.52	0	166^a^
AuYS_2_	23	2.95	3.68	0.62	0	166^a^
HgHfS_2_	14	0.79	1.34	—	2.4	166^b^
HgMnS_2_	−36	0	0	—	3.0	166^a^
HgPtS_2_	16	1.24	1.42	0.71	0	166^b^
**HgTiS**_2_	−14	0	0	—	0	1
HgZrS_2_	11	0.66	1.12	0.19	0	166^b^
BiAlS_2_	23	0	0	—	0	166^a^
BiCrS_2_	−7	0	0	—	3.0	166^a^
BiTiS_2_	6	0	0	—	0	166^a^
BiIrS_2_	−5	0	0	—	0	166^a^
InTiS_2_	−25	0	0	—	0	166^a^
InZrS_2_	9	0	0	—	0	164^a^
TlHfS_2_	−5	0	0	—	0	164^a^
**TlMnS**_2_	−33	0	0	—	2.6	166^a*^
TlTiS_2_	−21	0	0	—	0	164^a^
TlZrS_2_	−43	0	0	—	0	164^a^
SnCrS_2_	24	0	0	—	2.8	166^a^
SnTiS_2_	−4	0	0	—	0	166^a^
PbCrS_2_	25	0	0	—	2.8	166^a^
PbTiS_2_	−16	0	0	—	0	166^a^
PbZrS_2_	5	0	0	—	0	166^a^

For an explanation see caption of Table 2.

^*^The ground-state of TlMnS_2_ has space group number 15. The delafossite structure is only 10 meV/atom above this phase.

**Table 4:** Properties of the delafossite selenides ABSe_2_.

	*E*_hull_	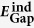	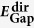	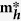	*μ*	Δ*E*_GS_
HMnSe_2_	−15	0	0	—	4.0	166^a*^
HScSe_2_	−15	2.28	2.51	0.97	0	11
KCrSe_2_	−83	0	0	—	3.0	166^b^
**KScSe**_**2**_	−157	2.37	3.11	0.98	0	166^b^
KRhSe_2_	−94	1.82	1.87	1.38	0	166^b^
KYSe_2_	−203	2.85	3.72	1.00	0	166^b^
RbScSe_2_	−141	2.40	3.13	1.03	0	166^b^
RbRhSe_2_	−80	1.78	1.92	1.26	0	166^b^
CsScSe_2_	−142	2.30	2.97	1.10	0	166^b^
CsLaSe_2_	−159	3.00	3.32	1.45	0	166^b^
CsRhSe_2_	−72	1.76	1.84	1.13	0	166^b^
CsYSe_2_	−206	2.74	3.45	1.10	0	166^b^
AgMnSe_2_	−54	0.64	0.84	—	4.0	166^b^
AgRhSe_2_	−1	0.97	1.73	0.30	0	166^a^
AuCoSe_2_	15	0.60	1.86	—	0	166^a^
AuCrSe_2_	2	0	0	—	3.0	166^a^
AuMnSe_2_	−44	0	0	—	4.0	166^a^
AuRhSe_2_	−13	1.01	1.99	0.08	0	166^a^
HgHfSe_2_	12	1.06	1.40	3.51	0	166^b^
HgMnSe_2_	−67	0	0	—	2.9	166^b^
HgNbSe_2_	0	0	0	—	0	38
HgPtSe_2_	14	1.28	1.66	0.90	0	166^b^
HgRhSe_2_	13	0	0	—	0	166^a^
HgTiSe_2_	1	0	0	—	0	1
HgVSe_2_	−7	0	0	—	0	6
HgZrSe_2_	11	0	0	1.46	0	166^b^
InZrSe_2_	17	0	0	—	0	164^b^
TlHfSe_2_	1	0	0	—	0	164^b^
**TlMnSe**_**2**_	−98	0	0	—	4.0	166^b^
TlRhSe_2_	−63	0	0	—	0	164^b^
TlTiSe_2_	23	0	0	—	0	164^b^
TlVSe_2_	−7	0	0	—	0	187
TlZrSe_2_	−40	0	0	—	0	164^b^
SbMnSe_2_	−4	0	0	—	4.0	11
BiCrSe_2_	17	0	0	—	3.0	166^a^
BiMnSe_2_	12	0	0	—	3.7	166^a^
BrNiSe_2_	−2	0	0	—	0	166^a†^
INiSe_2_	25	0	0	—	0	166^a^

For an explanation see caption of Table 2.

^*^The ground-state of HMnSe_2_ has space group number 8. The delafossite structure is 8 meV/atom above this phase.

^†^The ground-state of BrNiSe_2_ has space group number 2. The delafossite structure is 22 meV/atom above this phase.

**Table 5:** Properties of the delafossite tellurides ABTe_2_.

	*E*_hull_	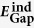	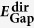	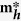	*μ*	Δ*E*_GS_
LiYTe_2_	−265	1.56	2.48	0.61	0	164^b^
NaYTe_2_	−314	1.89	2.94	0.84	0	166^b^
RbLaTe_2_	−108	2.30	2.87	1.10	0	166^b^
**CsBiTe**_**2**_	−68	1.57	1.75	1.18	0	166^b^
CsHfTe_2_	12	0	0	—	0	166^b^
CsScTe_2_	−70	1.64	2.20	0.77	0	166^b^
CsYTe_2_	−268	2.03	2.76	0.80	0	166^b^
BaCaTe_2_	25	2.26	2.94	0.58	0	166^b^
AgMnTe_2_	−4	0.01	0.28	—	4.0	166^b*^
HgHfTe_2_	10	0	0	—	0	166^b^
HgTiTe_2_	7	0	0	—	0	166^b^
HgVTe_2_	9	0	0	—	1.2	11
HgZrTe_2_	−65	0	0	0	0	166^b^
InYTe_2_	−169	0.50	1.44	—	0	166^b^
TlHfTe_2_	−3	0	0	—	0	164^b^
TlMnTe_2_	−47	0	0	—	4.0	164^b^
TlTiTe_2_	4	0	0	—	0.4	164^b^
**TlYTe**_**2**_	−230	1.20	2.05	0.24	0	166^b^
TlZrTe_2_	−59	0	0	—	0	164^b^
BrCoTe_2_	2	0	0	—	0	166^a^
BrIrTe_2_	−4	0.92	1.72	1.48	0	160
BrNiTe_2_	−30	0	0	—	0	166^b^
BrPdTe_2_	18	0	0	—	0	12
BrPtTe_2_	−68	0	0	—	0	166^b^
BrRhTe_2_	16	0	0	—	0	166^a^
INiTe_2_	8	0	0	—	0	13
IPdTe_2_	14	0	0	—	0	12
IPtTe_2_	−8	0	0	—	0	166^b^
IRhTe_2_	25	0	0	—	0	166^a^

For an explanation see caption of Table 2.

^*^The ground-state of HMnTe_2_ has space group number 72. The trigonal structure is 8 meV/atom above this phase.

## References

[b1] Kawazoe H. . P-type electrical conduction in transparent thin films of CuAlO_2_. Nature 389, 939–942, doi: 10.1038/40087 (1997).

[b2] Minami T. Transparent conducting oxide semiconductors for transparent electrodes. Semicond. Sci. Technol. 20, S35, doi: 10.1088/0268-1242/20/4/004 (2005).

[b3] Jain A. . The materials project: A materials genome approach to accelerating materials innovation. APL Mater. 1, 011002, doi: 10.1063/1.4812323 (2013).

[b4] Saal J. E., Kirklin S., Aykol M., Meredig B. & Wolverton C. Materials design and discovery with high-throughput density functional theory: the open quantum materials database (OQMD). JOM 65, 1501–1509, doi: 10.1007/s11837-013-0755-4 (2013).

[b5] Curtarolo S. . Aflowlib.org: A distributed materials properties repository from high-throughput ab initio calculations. Comput. Mater. Sci. 58, 227–235, doi: 10.1016/j.commatsci.2012.02.002 (2012).

[b6] Younsi M., Saadi S., Bouguelia A., Aider A. & Trari M. Synthesis and characterization of oxygen-rich delafossite CuYO_2+*x*_–Application to H_2_-photo production. Sol. Energy Mater. Sol. Cells 91, 1102–1109, doi: 10.1016/j.solmat.2007.03.014 (2007).

[b7] Saadi S., Bouguelia A., Derbal A. & Trari M. Hydrogen photoproduction over new catalyst CuLaO_2_. J. Photochem. Photobiol., A 187, 97–104, doi: 10.1016/j.jphotochem.2006.09.017 (2007).

[b8] Liu Y. . Luminescence of delafossite-type CuAlO_2_ fibers with Eu substitution for Al cations. Sci. Technol. Adv. Mater. 17, 200–209, doi: 10.1080/14686996.2016.1172024 (2016). 27877870 PMC5102038

[b9] Daniel U., Radu B. & Nicolae V. Photovoltaic performance of (Al, Mg)-doped CuCrO_2_ for p-type dye-sensitized solar cells application. Nanosci. Nanotechnol. 6, 71–76, doi: 10.5923/c.nn.201601.14 (2016).

[b10] Zakutayev A. Design of nitride semiconductors for solar energy conversion. J. Mater. Chem. A 4, 6742–6754, doi: 10.1039/C5TA09446A (2016).

[b11] Jiang T. . Copper borate as a photocathode in p-type dye-sensitized solar cells. RSC Adv. 6, 1549–1553, doi: 10.1039/C5RA24397A (2016).

[b12] Ono Y., i. Satoh K., Nozaki T. & Kajitani T. Structural, magnetic and thermoelectric properties of delafossite-type oxide, CuCr_1−*x*_Mg_*x*_O_2_ (0 < *x* < 0.05). In *25th International Conference on Thermoelectrics*, 2006, 92–96, doi: 10.1109/ICT.2006.331288 (2006).

[b13] Yasuhiro Ono T. N., Satoh Ken-ichi & Kajitani T. Structural, magnetic and thermoelectric properties of delafossite-type oxide, CuCr_1−*x*_Mg_*x*_O_2_ (0 ≤ *x* ≤ 0.05). Jpn. J. Appl. Phys. 46, 1071, doi: 10.1143/JJAP.46.1071 (2007).

[b14] Hayashi K., Nozaki T. & Kajitani T. Structure and high temperature thermoelectric properties of delafossite-type oxide CuFe_1−*x*_Ni_*x*_O_2_ (0 ≤ *x* ≤ 0.05). Jpn. J. Appl. Phys. 46, 5226, doi: 10.1143/JJAP.46.5226 (2007).

[b15] Ohkubo I. & Mori T. Anisotropic anomalies of thermoelectric transport properties and electronic structures in layered complex nitrides AMN_2_ (A = Na, Cu; M = Ta, Nb). Chem. Mater. 27, 7265–7275, doi: 10.1021/acs.chemmater.5b02015 (2015).

[b16] Chutirat Taddee T. K. & Amornkitbamrung V. Characterization of transparent superconductivity Fe-doped CuCrO_2_ delafossite oxide. Appl. Surf. Sci. 380, 237–242, doi: 10.1016/j.apsusc.2016.01.120 (2016).

[b17] Kushwaha P. . Nearly free electrons in a 5d delafossite oxide metal. Sci. Adv. 1, e1500692, doi: 10.1126/sciadv.1500692 (2015). 26601308 PMC4646822

[b18] Yanagi H., Kawazoe H., Kudo A., Yasukawa M. & Hosono H. Chemical design and thin film preparation of p-type conductive transparent oxides. J. Electroceram. 4, 407–414, doi: 10.1023/A:1009959920435 (2000).

[b19] Kawazoe H., Yanagi H., Ueda K. & Hosono H. Transparent p-type conducting oxides: Design and fabrication of p-n heterojunctions. MRS Bull. 25, 28–36, doi: 10.1557/mrs2000.148 (2000).

[b20] Marquardt M. A., Ashmore N. A. & Cann D. P. Crystal chemistry and electrical properties of the delafossite structure. Thin Solid Films 496, 146–156, doi: 10.1016/j.tsf.2005.08.316 (2006).

[b21] Scanlon D. O., Godinho K. G., Morgan B. J. & Watson G. W. Understanding conductivity anomalies in CuI-based delafossite transparent conducting oxides: Theoretical insights. J. Chem. Phys. 132, 024707, doi: 10.1063/1.3290815 (2010). 20095694

[b22] Scanlon D. O., Walsh A. & Watson G. W. Understanding the p-type conduction properties of the transparent conducting oxide CuBO_2_: A density functional theory analysis. Chem. Mater. 21, 4568–4576, doi: 10.1021/cm9015113 (2009).

[b23] Scanlon D. O. & Watson G. W. Understanding the p-type defect chemistry of CuCrO_2_. J. Mater. Chem. 21, 3655–3663, doi: 10.1039/C0JM03852K (2011).

[b24] Ginley D. S., Hosono H. & Paine D. C. (eds) Handbook of transparent conductors (Springer, 2010).

[b25] Han M. . Structural, electronic band transition and optoelectronic properties of delafossite CuGa_1−*x*_Cr_*x*_O_2_ (0 < *x* < 1) solid solution films grown by the sol-gel method. J. Mater. Chem. 22, 18463–18470, doi: 10.1039/C2JM33027J (2012).

[b26] Ueda K. . Epitaxial growth of transparent p-type conducting CuGaO_2_ thin films on sapphire (001) substrates by pulsed laser deposition. J. Appl. Phys. 89, 1790–1793, doi: 10.1063/1.1337587 (2001).

[b27] Yanagi H., Hase T., Ibuki S., Ueda K. & Hosono H. Bipolarity in electrical conduction of transparent oxide semiconductor CuInO_2_ with delafossite structure. Appl. Phys. Lett. 78, 1583–1585, doi: 10.1063/1.1355673 (2001).

[b28] Nagarajan R., Draeseke A., Sleight A. & Tate J. P-type conductivity in CuCr_1−*x*_Mg_*x*_O_2_ films and powders. J. Appl. Phys. 89, 8022–8025, doi: 10.1063/1.1372636 (2001).

[b29] Farrell L. . Synthesis of nanocrystalline Cu deficient CuCrO_2_–a high figure of merit p-type transparent semiconductor. J. Mater. Chem. C 4, 126–134, doi: 10.1039/C5TC03161C (2016).

[b30] Nagarajan R. . P-type conductivity in the delafossite structure. Int. J. Inorg. Mater. 3, 265–270, doi: 10.1063/1.1337587 (2001).

[b31] Wang Y., Gu Y., Wang T. & Shi W. Structural, optical and electrical properties of Mg-doped CuCrO_2_ thin films by sol-gel processing. J. Alloys Compd. 509, 5897–5902, doi: 10.1016/j.jallcom.2011.02.175 (2011).

[b32] Barnabé A., Thimont Y., Lalanne M., Presmanes L. & Tailhades P. p-type conducting transparent characteristics of delafossite Mg-doped CuCrO_2_ thin films prepared by rf-sputtering. J. Mater. Chem. C 3, 6012–6024, doi: 10.1039/C5TC01070E (2015).

[b33] Hosono H. Recent progress in transparent oxide semiconductors: Materials and device application. Thin Solid Films 515, 6000–6014, doi: 10.1016/j.tsf.2006.12.125 (2007).

[b34] Ueda K., Inoue S., Hirose S., Kawazoe H. & Hosono H. Transparent p-type semiconductor: LaCuOS layered oxysulfide. Appl. Phys. Lett. 77, 2701–2703, doi: 10.1063/1.1319507 (2000).

[b35] Hiramatsu H. . Degenerate p-type conductivity in wide-gap LaCuOS_1−*x*_Se_*x*_ (*x* = 0−1) epitaxial films. Appl. Phys. Lett. 82, 1048–1050, doi: 10.1063/1.1544643 (2003).

[b36] Krishnapriyan A., Barton P. T., Miao M. & Seshadri R. First-principles study of band alignments in the p-type hosts BaM_2_X_2_ (M = Cu, Ag; X = S, Se). J. Phys.: Condens. Matter 26, 155802, doi: 10.1088/0953-8984/26/15/155802 (2014). 24674946

[b37] Hautier G., Miglio A., Ceder G., Rignanese G.-M. & Gonze X. Identification and design principles of low hole effective mass p-type transparent conducting oxides. Nat. Commun. 4, 2292, doi: 10.1038/ncomms3292 (2013). 23939205 PMC3753546

[b38] Sarmadian N., Saniz R., Partoens B. & Lamoen D. Easily doped p-type, low hole effective mass, transparent oxides. Sci. Rep. 6, 20446, doi: 10.1038/srep20446 (2016). 26854336 PMC4745066

[b39] Cerqueira T. F. . Identification of novel Cu, Ag, and Au ternary oxides from global structural prediction. Chem. Mater. 27, 4562–4573, doi: 10.1021/acs.chemmater.5b00716 (2015).

[b40] Morgan D., Ceder G. & Curtarolo S. High-throughput and data mining with ab initio methods. Meas. Sci. Technol. 16, 296, doi: 10.1088/0957-0233/16/1/039 (2005).

[b41] Oganov A. R. (ed.) Modern Methods of Crystal Structure Prediction (Wiley-VCH, Berlin, 2010).

[b42] Glawe H., Sanna A., Gross E. K. U. & Marques M. A. L. The optimal one dimensional periodic table: a modified Pettifor chemical scale from data mining. New J. Phys. 18, 093011, doi: 10.1088/1367-2630/18/9/093011 (2016).

[b43] Pettifor D. A chemical scale for crystal-structure maps. Solid State Commun. 51, 31–34, doi: 10.1016/0038-1098(84)90765-8 (1984).

[b44] Sarmiento-Pérez R., Botti S. & Marques M. A. L. Optimized exchange and correlation semilocal functional for the calculation of energies of formation. J. Chem. Theory Comput. 11, 3844–3850, doi: 10.1021/acs.jctc.5b00529 (2015). 26574465

[b45] Takatsu H. . Magnetic structure of the conductive triangular-lattice antiferromagnet PdCrO_2_. Phys. Rev. B 89, 104408, doi: 10.1103/PhysRevB.89.104408 (2014).

[b46] Shannon R. D., Rogers D. B. & Prewitt C. T. Chemistry of noble metal oxides. I. Syntheses and properties of ABO_2_ delafossite compounds. Inorg. Chem. 10, 713–718, doi: 10.1021/ic50098a011 (1971).

[b47] Boon J. The crystal structure of NaBiS_2_ and KBiS_2_. Recl. Trav. Chim. Pays-Bas 63, 32–34, doi: 10.1002/recl.19440630203 (1944).

[b48] Havlak L., Fabry J., Henriques M. & Dušek M. Structure determination of KScS_2_, RbScS_2_ and KLnS_2_ (Ln = Nd, Sm, Tb, Dy, Ho, Er, Tm and Yb) and crystal chemical discussion. Acta Crystallogr., Sect. C: Struct. Chem. 71, 623–630, doi: 10.1016/0025-5408(87)90038-9 (2015). 26146403

[b49] Pachoud E. . Robustness of antiferromagnetism and pyroelectricity in AgCr_1−*x*_Rh_*x*_S_2_. Chem. Mater. 28, 1816–1822, doi: 10.1021/acs.chemmater.5b04948 (2016).

[b50] Ballestracci R. Une classe de nouveaux composés sulfurés de terres rares et d’argent de type AgTS_2_. C. R. Seances Acad. Sci., Ser. C 262, 1253–1256 (1966).

[b51] Sidorov M. . Structural investigation of mercury-intercalated titanium disulfide. 2. HRTEM of Hg_*x*_TiS_2_. Chem. Mater. 7, 1140–1152, doi: 10.1021/cm00054a014 (1995).

[b52] Veliev R., Sadykhov R., Asadov Y. G., Kerimova E. & Dzhabbarov A. Magnetization, paramagnetic susceptibility, and electrical conductivity of layered TIMnS_2_ and TIMnSe_2_ antiferromagnets. Crystallogr. Rep. 53, 130–133, doi: 10.1134/S1063774508010161 (2008).

[b53] Dijkstra J., Van Bruggen C., Haas C. & de Groot R. Electronic structure of the half-metallic ferromagnet KCrSe_2_. Phys. Rev. B 40, 7973, doi: 10.1103/PhysRevB.40.7973 (1989). 9991230

[b54] Trippel A., Lazarev V. & Berul S. Synthesis and properties of some compounds ABiTe_2_. Zh. Neorg. Khim. 23, 707–710 (1978).

[b55] Kabré S. M. G. & Julien Pouzol, M. Sur une nouvelle famille de tellurures doubles de thallium(I) et de terres rares. C. R. Seances Acad. Sci., Ser. C 275, 1367–1370 (1972).

[b56] Heyd J., Scuseria G. E. & Ernzerhof M. Hybrid functionals based on a screened coulomb potential. J. Chem. Phys **118**, 8207–8215, doi: 10.1063/1.1564060 (2003).

[b57] Vidal J., Trani F., Bruneval F., Marques M. A. L. & Botti S. Effects of electronic and lattice polarization on the band structure of delafossite transparent conductive oxides. Phys. Rev. Lett. 104, 136401, doi: 10.1103/PhysRevLett.104.136401 (2010). 20481897

[b58] Trani F., Vidal J., Botti S. & Marques M. A. L. Band structures of delafossite transparent conductive oxides from a self-consistent *GW* approach. Phys. Rev. B 82, 085115, doi: 10.1103/PhysRevB.82.085115 (2010). 20481897

[b59] Pellicer-Porres J. . On the band gap of CuAlO_2_ delafossite. Appl. Phys. Lett. 88, 181904–181904, doi: 10.1063/1.2200398 (2006).

[b60] Kresse G. & Furthmüller J. Efficiency of ab-initio total energy calculations for metals and semiconductors using a plane-wave basis set. Comput. Mater. Sci. 6, 15–50, doi: 10.1016/0927-0256(96)00008-0 (1996). 9984901

[b61] Kresse G. & Furthmüller J. Efficient iterative schemes for ab initio total-energy calculations using a plane-wave basis set. Phys. Rev. B 54, 11169–11186, doi: 10.1103/PhysRevB.54.11169 (1996). 9984901

[b62] Blöchl P. E. Projector augmented-wave method. Phys. Rev. B 50, 17953, doi: 10.1103/PhysRevB.50.17953 (1994). 9976227

[b63] Perdew J. P., Burke K. & Ernzerhof M. Generalized gradient approximation made simple. Phys. Rev. Lett. 77, 3865–3868, doi: 10.1103/PhysRevLett.77.3865 (1996). 10062328

[b64] Tran F., Stelzl J. & Blaha P. Rungs 1 to 4 of DFT Jacob’s ladder: Extensive test on the lattice constant, bulk modulus, and cohesive energy of solids. The Journal of Chemical Physics 144, doi: 10.1063/1.4948636 (2016). 27250292

[b65] Ong S. P. . Python materials genomics (pymatgen): A robust, open-source python library for materials analysis. Comput. Mater. Sci. 68, 314–319, doi: 10.1016/j.commatsci.2012.10.028 (2013).

[b66] Bergerhoff G. & Brown I. Inorganic Crystal Structure Database (International Union of Crystallography, Chester, 1987).

[b67] Belsky A., Hellenbrandt M., Karen V. L. & Luksch P. New developments in the Inorganic Crystal Structure Database (ICSD): accessibility in support of materials research and design. Acta Crystallogr., Sect. B: Struct. Sci., Cryst. Eng. Mater. 58, 364–369, doi: 10.1107/S0108768102006948 (2002). 12037357

[b68] Oganov A. R. & Glass C. W. Crystal structure prediction using ab initio evolutionary techniques: Principles and applications. J. Chem. Phys. 124, 244704, doi: 10.1063/1.2210932 (2006). 16821993

[b69] Wang Y., Lv J., Zhu L. & Ma Y. Crystal structure prediction via particle-swarm optimization. Phys. Rev. B 82, 094116, doi: 10.1103/PhysRevB.82.094116 (2010).

[b70] Wang Y., Lv J., Zhu L. & Ma Y. Calypso: A method for crystal structure prediction. Comput. Phys. Commun. 183, 2063–2070, doi: 10.1016/j.cpc.2012.05.008 (2012).

[b71] Pickard C. J. & Needs R. Ab initio random structure searching. J. Phys.: Condens. Matter 23, 053201, doi: 10.1063/1.2210932 (2011). 21406903

[b72] Goedecker S. Minima hopping: An efficient search method for the global minimum of the potential energy surface of complex molecular systems. J. Chem. Phys. 120, 9911–9917, doi: 10.1063/1.1724816 (2004). 15268009

[b73] Amsler M. & Goedecker S. Crystal structure prediction using the minima hopping method. J. Chem. Phys. 133, 224104, doi: 10.1063/1.3512900 (2010). 21171680

[b74] Amsler M. . Crystal structure of cold compressed graphite. Phys. Rev. Lett. 108, 065501, doi: 10.1103/PhysRevLett.108.065501 (2012). 22401083

[b75] Flores-Livas J. A. . High-pressure structures of disilane and their superconducting properties. Phys. Rev. Lett. 108, 117004, doi: 10.1103/PhysRevLett.108.117004 (2012). 22540502

[b76] Huan T. D. . Low-energy polymeric phases of alanates. Phys. Rev. Lett. 110, 135502, doi: 10.1103/PhysRevLett.110.135502 (2013). 23581335

[b77] Amsler M., Botti S., Marques M. A. L. & Goedecker S. Conducting boron sheets formed by the reconstruction of the *α*-boron (111) surface. Phys. Rev. Lett. 111, 136101, doi: 10.1103/PhysRevLett.110.135502 (2013). 24116795

[b78] Sarmiento-Perez R., Cerqueira T. F. T., Körbel S., Botti S. & Marques M. A. L. Prediction of stable nitride perovskites. Chem. Mater. 27, 5957–5963, doi: 10.1021/acs.chemmater.5b02026 (2015).

[b79] Madsen G. K. & Singh D. J. BoltzTraP. A code for calculating band-structure dependent quantities. Comput. Phys. Commun. 175, 67–71, doi: 10.1016/j.cpc.2006.03.007 (2006).

[b80] Schleife A., Fuchs F., Rödl C., Furthmüller J. & Bechstedt F. Branch-point energies and band discontinuities of III-nitrides and III-II-oxides from quasiparticle band-structure calculation. Appl. Phys. Lett. 94, 012104, doi: 10.1063/1.3059569 (2009).

[b81] Robertson J. & Clark S. J. Limits to doping in oxides. Phys. Rev. B 83, 075205, doi: 10.1103/PhysRevB.83.075205 (2011).

[b82] Momma K. & Izumi F. Vesta 3 for three-dimensional visualization of crystal, volumetric and morphology data. J. Appl. Crystallogr. 44, 1272–1276, doi: 10.1107/S0021889811038970 (2011).

