# Supplementary material for: High-throughput search of ternary chalcogenides for p-type transparent electrodes
Source: Sci Rep. 2017 Mar 7;7:43179. doi: 10.1038/srep43179 (PMC5339873; doi:10.1038/srep43179)
Supplement: Supplementary Information [file srep43179-s2.pdf]

# Supplementary Information for "High-throughput search of ternary chalcogenides for p-type transparent electrodes"

Jingming Shi[1], Tiago F.T. Cerqueira[2], Wenwen Cui[1],
Fernando Nogueira[4],Silvana Botti[2,5], Miguel A.L. Marques[3,5]

[1]Institut Lumière Matière (UMR5306), Université Lyon 1-CNRS,
Université de Lyon, F-69622 Villeurbanne Cedex, France
[2]Institut für Festkörpertheorie und -optik, Friedrich-Schiller-Universität Jena,
Max-Wien-Platz 1, 07743 Jena, Germany
[3]Institut für Physik, Martin-Luther-Universität Halle-Wittenberg,
D-06099 Halle, Germany
[4]CFC, Departamento de Física, Universidade de Coimbra,
3004-516 Coimbra, Portugal
[5]European Theoretical Spectroscopy Facility

December 8, 2016

Table S1: Structual parameters of the new stable delafossite and closely related phases with composition $ABO_2$.

| Compounds | Spg nunmber (name) | Lattice parameters | Coordinates parameters |
|---|---|---|---|
| $KInO_2$ | $166^b$(R -3 2/m) | a=3.35949 Å<br>b=3.35949 Å<br>c=18.54410 Å<br>$\alpha$=$\beta$=90°,$\gamma$=120° | In 0.00000 0.00000 0.50000<br>K 0.00000 0.00000 0.00000<br>O 0.00000 0.00000 -0.22894 |
| $RbInO_2$ | $166^b$(R -3 2/m) | a=3.39981 Å<br>b=3.39981 Å<br>c=19.36914 Å<br>$\alpha$=$\beta$=90°,$\gamma$=120° | In 0.00000 0.00000 0.00000<br>Rb 0.00000 0.00000 0.50000<br>O 0.00000 0.00000 -0.27463 |
| $RbRhO_2$ | $166^b$(R -3 2/m) | a=3.29582 Å<br>b=3.29582 Å<br>c=17.953 Å<br>$\alpha$=$\beta$=90°,$\gamma$=120° | Rh 0.00000 0.00000 0.00000<br>Rb 0.00000 0.00000 0.50000<br>O 0.00000 0.00000 -0.28092 |
| $CsLaO_2$ | $166^b$(R -3 2/m) | a=3.78465 Å<br>b=3.78465 Å<br>c=20.84 Å<br>$\alpha$=$\beta$=90°,$\gamma$=120° | La 0.00000 0.00000 0.00000<br>Cs 0.00000 0.00000 0.50000<br>O 0.00000 0.00000 -0.27684 |
| $MgNiO_2$ | $166^b$(R -3 2/m) | a=3.00143 Å<br>b=3.00143 Å<br>c=14.558 Å<br>$\alpha$=$\beta$=90°,$\gamma$=120° | Ni 0.00000 0.00000 0.50000<br>Mg 0.00000 0.00000 0.00000<br>O 0.00000 0.00000 0.25082 |
| $PdAlO_2$ | $166^a$(R -3 2/m) | a=2.86607 Å<br>b=2.86607 Å<br>c=18.09580 Å<br>$\alpha$=$\beta$=90°,$\gamma$=120° | Pd 0.00000 0.00000 0.50000<br>Al 0.00000 0.00000 0.0000<br>O 0.00000 0.00000 -0.38662 |
| $PdCrO_2$ | $166^a$(R -3 2/m) | a=3.00903 Å<br>b=3.00903 Å<br>c=18.23102 Å<br>$\alpha$=$\beta$=90°,$\gamma$=120° | Pd 0.00000 0.00000 0.50000<br>Cr 0.00000 0.00000 0.00000<br>O 0.00000 0.00000 -0.38882 |
| $PdRhO_2$ | $166^a$(R -3 2/m) | a=3.08303 Å<br>b=3.08303 Å<br>c=18.30676 Å<br>$\alpha$=$\beta$=90°,$\gamma$=120° | Pd 0.00000 0.00000 0.00000<br>Rh 0.00000 0.00000 0.50000<br>O 0.00000 0.00000 0.11048 |
| $PtAlO_2$ | $166^a$(R -3 2/m) | a=2.85681 Å<br>b=2.85681 Å<br>c=18.15331 Å<br>$\alpha$=$\beta$=90°,$\gamma$=° | Pt 0.00000 0.00000 0.00000<br>Al 0.00000 0.00000 0.50000<br>O 0.00000 0.00000 0.11357 |
| $PtNiO_2$ | $166^a$(R -3 2/m) | a=2.92246 Å<br>b=2.92246 Å<br>c=18.21565 Å<br>$\alpha$=$\beta$=90°,$\gamma$=120° | Pt 0.00000 0.00000 0.50000<br>Ni 0.00000 0.00000 0.00000<br>O 0.00000 0.00000 -0.39247 |

Table S2: Structual parameters of the new stable delafossite and closely related phases with composition $ABO_2$. Continuation of Table S1.

| | | | |
|---|---|---|---|
| $HgMgO_2$ | $166^a$(R -3 2/m) | a=3.25902 Å<br>b=3.25902 Å<br>c=18.25154 Å<br>$\alpha$=$\beta$=90°,$\gamma$=120° | Hg 0.00000 0.00000 0.00000<br>Mg 0.00000 0.00000 0.50000<br>O 0.00000 0.00000 0.11034 |
| $TlRhO_2$ | $166^a$(R -3 2/m) | a=3.16242 Å<br>b=3.16242 Å<br>c=20.77487 Å<br>$\alpha$=$\beta$=90°,$\gamma$=120° | Tl 0.00000 0.00000 0.00000<br>Rh 0.00000 0.00000 0.50000<br>O 0.00000 0.00000 -0.12102 |
| $BrCdO_2$ | $166^a$(R -3 2/m) | a=3.51303 Å<br>b=3.51303 Å<br>c=18.64577 Å<br>$\alpha$=$\beta$=90°,$\gamma$=120° | Cd 0.00000 0.00000 0.00000<br>Br 0.00000 0.00000 0.50000<br>O 0.00000 0.00000 0.39411 |
| $BrLaO_2$ | 12 (A 1 2/m 1) | a=8.25488 Å<br>b=3.94162 Å<br>c=6.85559 Å<br>$\alpha$=$\gamma$=90°,$\beta$=134.99° | La 0.000 0.000 0.000<br>Br 0.500 0.500 0.500<br>O -0.160 0.000 1.000 |
| $BrNiO_2$ | $166^a$(R -3 2/m) | a=3.11474 Å<br>b=3.11474 Å<br>c=17.78526 Å<br>$\alpha$=$\beta$=90°,$\gamma$=120° | Br 0.00000 0.00000 0.00000<br>Ni 0.00000 0.00000 0.50000<br>O 0.00000 0.00000 -0.11013 |
| $BrTlO_2$ | $166^a$(R -3 2/m) | a=3.63606 Å<br>b=3.63606 Å<br>c=18.94165 Å<br>$\alpha$=$\beta$=90°,$\gamma$=120° | Tl 0.00000 0.00000 0.50000<br>Br 0.00000 0.00000 0.00000<br>O 0.00000 0.00000 -0.11016 |

Table S3: Structual parameters of the new stable delafossite and closely related phases with composition $ABS_2$.

| | | | |
|---|---|---|---|
| $HIrS_2$ | 160 (R 3 m) | a=3.65516 Å<br>b=3.65516 Å<br>c=17.47866 Å<br>$\alpha$=$\beta$=90°,$\gamma$=120° | Ir 0.000 0.000 0.191<br>S 0.000 0.000 -0.210<br>S 0.000 0.000 -0.416<br>H 0.000 0.000 -0.335 |
| $KBiS_2$ | $166^b$(R -3 2/m) | a=4.15325 Å<br>b=4.15325 Å<br>c=22.36348 Å<br>$\alpha$=$\beta$=90°,$\gamma$=120° | Bi 0.00000 0.00000 0.00000<br>K 0.00000 0.00000 0.50000<br>S 0.00000 0.00000 -0.26288 |
| $KScS_2$ | $166^b$(R -3 2/m) | a=3.84107 Å<br>b=3.84107 Å<br>c=21.87898 Å<br>$\alpha$=$\beta$=90°,$\gamma$=120° | Sc 0.00000 0.00000 0.00000<br>K 0.00000 0.00000 0.50000<br>S 0.00000 0.00000 -0.26922 |
| $KYS_2$ | $166^b$(R -3 2/m) | a=4.05568 Å<br>b=4.05568 Å<br>c=22.12079 Å<br>$\alpha$=$\beta$=90°,$\gamma$=120° | Y 0.00000 0.00000 0.00000<br>K 0.00000 0.00000 0.50000<br>S 0.00000 0.00000 -0.26596 |
| $RbScS_2$ | $166^b$(R -3 2/m) | a=3.87064 Å<br>b=3.87064 Å<br>c=22.80286 Å<br>$\alpha$=$\beta$=90°,$\gamma$=120° | Rb 0.00000 0.00000 0.00000<br>Sc 0.00000 0.00000 0.50000<br>S 0.00000 0.00000 0.22769 |
| $AgCoS_2$ | $166^a$(R -3 2/m) | a=3.35973 Å<br>b=3.35973 Å<br>c=21.26877 Å<br>$\alpha$=$\beta$=°,$\gamma$=120° | Ag 0.00000 0.00000 0.00000<br>Co 0.00000 0.00000 0.50000<br>S 0.00000 0.00000 -0.11332 |
| $AgMnS_2$ | $166^a$(R -3 2/m) | a=3.32591 Å<br>b=3.32591 Å<br>c=22.33769 Å<br>$\alpha$=$\beta$=90°,$\gamma$=120° | Ag 0.00000 0.00000 0.00000<br>Mn 0.00000 0.00000 0.50000<br>S 0.00000 0.00000 -0.10862 |
| $AgRhS_2$ | $166^a$(R -3 2/m) | a=3.57217 Å<br>b=3.57217 Å<br>c=21.49311 Å<br>$\alpha$=$\beta$=90°,$\gamma$=120° | Ag 0.00000 0.00000 0.00000<br>Rh 0.00000 0.00000 0.50000<br>S 0.00000 0.00000 -0.11138 |
| $AgScS_2$ | 160 (R 3 m) | a=b=3.76128 Å<br>c=20.68221 Å<br>$\alpha$=$\beta$=90°<br>$\gamma$=120° | Ag 0.00 0.00 0.00658<br>Sc 0.00 0.00 0.15680<br>S 0.00 0.00 0.417<br>S 0.00 0.00 -0.11144 |
| $AgIrS_2$ | $166^a$(R -3 2/m) | a=3.60138 Å<br>b=3.60138 Å<br>c=21.37937 Å<br>$\alpha$=$\beta$=90°,$\gamma$=120° | Ir 0.00000 0.00000 0.50000<br>Ag 0.00000 0.00000 0.00000<br>S 0.00000 0.00000 -0.11235 |

Table S4: Structual parameters of the new stable delafossite and closely related phases with composition $ABS_2$. Continuation of Table S3.

| | | | |
|---|---|---|---|
| $AgYS_2$ | 156 (P 3 m 1) | a=b=4.05641 Å<br>c=6.63987 Å<br>$\alpha$=$\beta$=90°<br>$\gamma$=120° | Ag 0.333 0.667 -0.421<br>Y 0.00 0.00 -0.00178<br>S 0.333 0.667 0.20456<br>S 0.667 0.333 -0.23684 |
| $AuAlS_2$ | $166^{a}$(R -3 2/m) | a=3.56258 Å<br>b=3.56258 Å<br>c=21.56252 Å<br>$\alpha$=$\beta$=90°,$\gamma$=120° | Au 0.00000 0.00000 0.00000<br>S 0.00000 0.00000 -0.10762<br>Al 0.00000 0.00000 0.50000 |
| $AuBiS_2$ | $166^{a}$(R -3 2/m) | a=4.28266 Å<br>b=4.28266 Å<br>c=22.26884 Å<br>$\alpha$=$\beta$=90°,$\gamma$=120° | Bi 0.00000 0.00000 0.50000<br>Au 0.00000 0.00000 0.0000<br>S 0.00000 0.00000 -0.10296 |
| $AuCoS_2$ | $166^{a}$(R -3 2/m) | a=3.36726 Å<br>b=3.36726 Å<br>c=20.78827 Å<br>$\alpha$=$\beta$=90°,$\gamma$=120° | Au 0.00000 0.00000 0.00000<br>Co 0.00000 0.00000 0.50000<br>S 0.00000 0.00000 -0.11258 |
| $AuInS_2$ | $166^{a}$(R -3 2/m) | a=3.94181 Å<br>b=3.94181 Å<br>c=22.00386 Å<br>$\alpha$=$\beta$=90°,$\gamma$=120° | Au 0.00000 0.00000 0.00000<br>In 0.00000 0.00000 0.50000<br>S 0.00000 0.00000 -0.10468 |
| $AuIrS_2$ | $166^{a}$(R -3 2/m) | a=3.61618 Å<br>b=3.61618 Å<br>c=20.86395 Å<br>$\alpha$=$\beta$=90°,$\gamma$=120° | Au 0.00000 0.00000 0.00000<br>Ir 0.00000 0.00000 0.50000<br>S 0.00000 0.00000 -0.11166 |
| $AuMnS_2$ | $166^{a}$(R -3 2/m) | a=3.33532 Å<br>b=3.33532 Å<br>c=21.81510 Å<br>$\alpha$=$\beta$=90°,$\gamma$=120° | Au 0.00000 0.00000 0.00000<br>Mn 0.00000 0.00000 0.50000<br>S 0.00000 0.00000 -0.10786 |
| $AuRhS_2$ | $166^{a}$(R -3 2/m) | a=3.58538 Å<br>b=3.58538 Å<br>c=20.98328 Å<br>$\alpha$=$\beta$=90°,$\gamma$=120° | Au 0.00000 0.00000 0.00000<br>Rh 0.00000 0.00000 0.50000<br>S 0.00000 0.00000 -0.11063 |
| $AuScS_2$ | $166^{a}$(R -3 2/m) | a=3.82016 Å<br>b=3.82016 Å<br>c=22.01038 Å<br>$\alpha$=$\beta$=90°,$\gamma$=120° | Au 0.00000 0.00000 0.00000<br>Sc 0.00000 0.00000 0.50000<br>S 0.00000 0.00000 -0.10493 |
| $AuYS_2$ | $166^{a}$(R -3 2/m) | a=4.13622 Å<br>b=4.13622 Å<br>c=22.10901 Å<br>$\alpha$=$\beta$=90°,$\gamma$=120° | Au 0.00000 0.00000 0.00000<br>Y 0.00000 0.00000 0.50000<br>S 0.00000 0.00000 -0.10430 |
| $HgHfS_2$ | $166^{b}$(R -3 2/m) | a=3.63137 Å<br>b=3.63137 Å<br>c=28.69144 Å<br>$\alpha$=$\beta$=90°,$\gamma$=120° | Hg 0.00000 0.00000 0.00000<br>Hf 0.00000 0.00000 0.50000<br>S 0.00000 0.00000 0.21733 |

Table S5: Structual parameters of the new stable delafossite and closely related phases with composition $ABS_2$. Continuation of Table S4.

| | | | |
|---|---|---|---|
| $HgMnS_2$ | $166^a$(R -3 2/m) | a=3.34606 Å<br>b=3.34606 Å<br>c=27.51030 Å<br>$\alpha=\beta=90°,\gamma=120°$ | Hg 0.00000 0.00000 0.00000<br>Mn 0.00000 0.00000 0.50000<br>S 0.00000 0.00000 -0.11876 |
| $HgPtS_2$ | $166^b$(R -3 2/m) | a=3.57244 Å<br>b=3.57244 Å<br>c=26.37048 Å<br>$\alpha=\beta=90°,\gamma=120°$ | Hg 0.00000 0.00000 0.50000<br>Pt 0.00000 0.00000 0.00000<br>S 0.00000 0.00000 -0.28647 |
| $HgTiS_2$ | 1 (P 1) | a=5.90492 Å<br>b=3.39530 Å<br>c=9.60591 Å<br>$\alpha=87.44096°$<br>$\beta=105.41234°$<br>$\gamma=89.99510°$ | Hg -0.44390 -0.49609 1.00000<br>Hg 0.48651 -0.42345 -0.49379<br>Ti -0.01571 -0.22243 0.00483<br>Ti 0.48495 0.28364 0.00500<br>S -0.11507 0.26144 0.16022<br>S 0.08440 0.30054 -0.14989<br>S 0.38464 -0.23908 0.15946<br>S -0.41558 -0.19991 -0.15015 |
| $HgZrS_2$ | $166^b$(R -3 2/m) | a=3.67081 Å<br>b=3.67081 Å<br>c=28.05292 Å<br>$\alpha=\beta=90°,\gamma=120°$ | Hg 0.00000 0.00000 0.50000<br>Zr 0.00000 0.00000 0.00000<br>S 0.00000 0.00000 -0.28090 |
| $BiAlS_2$ | $166^a$(R -3 2/m) | a=3.53328 Å<br>b=3.53328 Å<br>c=24.70228 Å<br>$\alpha=\beta=90°,\gamma=120°$ | Bi 0.00000 0.00000 0.00000<br>S 0.00000 0.00000 -0.11385<br>Al 0.00000 0.00000 0.50000 |
| $BiCrS_2$ | $166^a$(R -3 2/m) | a=3.52727 Å<br>b=3.52727 Å<br>c=24.57201 Å<br>$\alpha=\beta=90°,\gamma=120°$ | Bi 0.00000 0.00000 0.00000<br>Cr 0.00000 0.00000 0.50000<br>S 0.00000 0.00000 -0.11492 |
| $BiTiS_2$ | $166^a$(R -3 2/m) | a=3.44094 Å<br>b=3.44094 Å<br>c=25.83420 Å<br>$\alpha=\beta=90°,\gamma=120°$ | Bi 0.00000 0.00000 0.50000<br>Ti 0.00000 0.00000 0.00000<br>S 0.00000 0.00000 -0.38896 |
| $BiIrS_2$ | $166^a$(R -3 2/m) | a=3.59387 Å<br>b=3.59387 Å<br>c=24.25913 Å<br>$\alpha=\beta=90°,\gamma=120°$ | Bi 0.00000 0.00000 0.00000<br>Ir 0.00000 0.00000 0.50000<br>S 0.00000 0.00000 -0.11851 |
| $InTiS_2$ | $166^a$(R -3 2/m) | a=3.39205 Å<br>b=3.39205 Å<br>c=26.02497 Å<br>$\alpha=\beta=90°,\gamma=120°$ | In 0.00000 0.00000 0.00000<br>Ti 0.00000 0.00000 0.50000<br>S 0.00000 0.00000 -0.11113 |
| $InZrS_2$ | 164 (P -3 2/m 1) | a=b=3.61485 Å<br>c=8.18948 Å<br>$\alpha=\beta=90°,\gamma=120°$ | In 0.00000 0.00000 0.50000<br>Zr 0.00000 0.00000 0.00000<br>S 0.33333 0.66667 0.18891 |

Table S6: Structual parameters of the new stable delafossite and closely related phases with composition $ABS_2$. Continuation of Table S5.

| | | | |
|---|---|---|---|
| $TlHfS_2$ | 164 (P -3 2/m 1) | a=b=3.59750 Å<br>c=8.54200 Å<br>$\alpha$=$\beta$=90°,$\gamma$=120° | Tl 0.00000 0.00000 0.00000<br>Hf 0.00000 0.00000 0.50000<br>S 0.33333 0.66667 -0.32445 |
| $TlMnS_2$ | 166[a](R -3 2/m) | a=3.37108 Å<br>b=3.37108 Å<br>c=26.36703 Å<br>$\alpha$=$\beta$=90°,$\gamma$=120° | Tl 0.00000 0.00000 0.00000<br>Mn 0.00000 0.00000 0.50000<br>S 0.00000 0.00000 -0.11764 |
| $TlTiS_2$ | 164 (P -3 2/m 1) | a=b=3.47225 Å<br>c=8.17414 Å<br>$\alpha$=$\beta$=90°,$\gamma$=120° | Tl 0.00000 0.00000 0.00000<br>Ti 0.00000 0.00000 0.50000<br>S 0.33333 0.66667 -0.32576 |
| $TlZrS_2$ | 164 (P -3 2/m 1) | a=b=3.64723 Å<br>c=8.25608 Å<br>$\alpha$=$\beta$=90°,$\gamma$=120° | Tl 0.00000 0.00000 0.00000<br>Zr 0.00000 0.00000 0.50000<br>S 0.33333 0.66667 0.31475 |
| $SnCrS_2$ | 166[a](R -3 2/m) | a=3.46684 Å<br>b=3.46684 Å<br>c=24.44877 Å<br>$\alpha$=$\beta$=90°,$\gamma$=120° | Sn 0.00000 0.00000 0.00000<br>Cr 0.00000 0.00000 0.50000<br>S 0.00000 0.00000 -0.11396 |
| $SnTiS_2$ | 166[a](R -3 2/m) | a=3.39805 Å<br>b=3.39805 Å<br>c=25.67830 Å<br>$\alpha$=$\beta$=90°,$\gamma$=120° | Sn 0.00000 0.00000 0.00000<br>Ti 0.00000 0.00000 0.50000<br>S 0.00000 0.00000 -0.11020 |
| $PbCrS_2$ | 166[a](R -3 2/m) | a=3.46683 Å<br>b=3.46683 Å<br>c=25.11917 Å<br>$\alpha$=$\beta$=90°,$\gamma$=120° | Pb 0.00000 0.00000 0.00000<br>Cr 0.00000 0.00000 0.50000<br>S 0.00000 0.00000 -0.11541 |
| $PbTiS_2$ | 166[a](R -3 2/m) | a=3.43824 Å<br>b=3.43824 Å<br>c=26.24558 Å<br>$\alpha$=$\beta$=90°,$\gamma$=120° | Pb 0.00000 0.00000 0.00000<br>Ti 0.00000 0.00000 0.50000<br>S 0.00000 0.00000 -0.11203 |
| $PbZrS_2$ | 166[a](R -3 2/m) | a=3.60049 Å<br>b=3.60049 Å<br>c=26.99142 Å<br>$\alpha$=$\beta$=90°,$\gamma$=120° | Pb 0.00000 0.00000 0.00000<br>Zr 0.00000 0.00000 0.50000<br>S 0.00000 0.00000 -0.10959 |

Table S7: Structual parameters of the new stable delafossite and closely related phases with composition $ABSe_2$.

| Compounds | Spg nunmber (name) | Lattice parameters | Coordinates parameters |
|---|---|---|---|
| $HMnSe_2$ | $166^a$(R -3 2/m) | a=3.81064 Å<br>b=3.81064 Å<br>c=18.66232 Å<br>$\alpha=\beta=90°,\gamma=120°$ | Se 0.00000 0.00000 -0.09441<br>Mn 0.00000 0.00000 0.50000<br>H 0.00000 0.00000 0.00000 |
| $HScSe_2$ | 11 (P 1 21/m 1) | a=7.00059 Å<br>b=3.97127 Å<br>c=7.02455 Å<br>$\alpha=\gamma=90°,\beta=108°$ | Se -0.34909 0.25000 -0.26884<br>Se -0.14044 0.25000 0.30354<br>Sc 0.27769 0.25000 0.49027<br>H -0.22547 0.25000 0.07693 |
| $KCrSe_2$ | $166^b$(R -3 2/m) | a=3.86391 Å<br>b=3.86391 Å<br>c=22.19740 Å<br>$\alpha=\beta=90°,\gamma=120°$ | Se 0.00000 0.00000 0.27399<br>Cr 0.00000 0.00000 0.00000<br>K 0.00000 0.00000 0.50000 |
| $KScSe_2$ | $166^b$(R -3 2/m) | a=4.01205 Å<br>b=4.01205 Å<br>c=22.83015 Å<br>$\alpha=\beta=90°,\gamma=120°$ | Se 0.00000 0.00000 0.26816<br>Sc 0.00000 0.00000 0.00000<br>K 0.00000 0.00000 0.50000 |
| $KRhSe_2$ | $166^b$(R -3 2/m) | a=3.90273 Å<br>b=3.90273 Å<br>c=21.30640 Å<br>$\alpha=\beta=90°,\gamma=120°$ | Rh 0.00000 0.00000 0.00000<br>Se 0.00000 0.00000 0.27722<br>K 0.00000 0.00000 0.50000 |
| $KYSe_2$ | $166^b$(R -3 2/m) | a=4.21753 Å<br>b=4.21753 Å<br>c=23.04535 Å<br>$\alpha=\beta=90°,\gamma=120°$ | Y 0.00000 0.00000 0.50000<br>Se 0.00000 0.00000 0.23517<br>K 0.00000 0.00000 0.00000 |
| $RbScSe_2$ | $166^b$(R -3 2/m) | a=4.04394 Å<br>b=4.04394 Å<br>c=23.74805 Å<br>$\alpha=\beta=90°,\gamma=120°$ | Rb 0.00000 0.00000 0.50000<br>Se 0.00000 0.00000 0.27115<br>Sc 0.00000 0.00000 0.00000 |
| $RbRhSe_2$ | $166^b$(R -3 2/m) | a=3.93388 Å<br>b=3.93388 Å<br>c=22.07918 Å<br>$\alpha=\beta=90°,\gamma=120°$ | Rh 0.00000 0.00000 0.00000<br>Rb 0.00000 0.00000 0.50000<br>Se 0.00000 0.00000 0.28001 |
| $CsScSe_2$ | $166^b$(R -3 2/m) | a=4.07381 Å<br>b=4.07381 Å<br>c=24.81908 Å<br>$\alpha=\beta=90°,\gamma=120°$ | Cs 0.00000 0.00000 0.50000<br>Se 0.00000 0.00000 0.27437<br>Sc 0.00000 0.00000 0.00000 |
| $CsLaSe_2$ | $166^b$(R -3 2/m) | a=4.50022 Å<br>b=4.50022 Å<br>c=25.13292 Å<br>$\alpha=\beta=90°,\gamma=120°$ | La 0.00000 0.00000 0.00000<br>Cs 0.00000 0.00000 0.50000<br>Se 0.00000 0.00000 0.26978 |
| $CsRhSe_2$ | $166^b$(R -3 2/m) | a=3.97311 Å<br>b=3.97311 Å<br>c=22.88826 Å<br>$\alpha=\beta=90°,\gamma=120°$ | Cs 0.00000 0.00000 0.00000<br>Rh 0.00000 0.00000 0.50000<br>Se 0.00000 0.00000 0.21710 |

Table S8: Structual parameters of the new stable delafossite and closely related phases with composition $ABSe_2$. Continuation of Table S7.

| | | | |
|---|---|---|---|
| $CsYSe_2$ | $166^b$(R -3 2/m) | a=4.27003 Å<br>b=4.27003 Å<br>c=25.11956 Å<br>$\alpha=\beta=90°,\gamma=120°$ | Cs 0.00000 0.00000 0.00000<br>Y 0.00000 0.00000 0.50000<br>Se 0.00000 0.00000 -0.22865 |
| $AgMnSe_2$ | $166^b$(R -3 2/m) | a=3.76196 Å<br>b=3.76196 Å<br>c=19.82880 Å<br>$\alpha=\beta=90°,\gamma=120°$ | Ag 0.00000 0.00000 0.50000<br>Se 0.00000 0.00000 -0.26018<br>Mn 0.00000 0.00000 0.00000 |
| $AgRhSe_2$ | $166^a$(R -3 2/m) | a=3.75170 Å<br>b=3.75170 Å<br>c=22.62660 Å<br>$\alpha=\beta=90°,\gamma=120°$ | Ag 0.00000 0.00000 0.00000<br>Rh 0.00000 0.00000 0.50000<br>Se 0.00000 0.00000 -0.11125 |
| $AuCoSe_2$ | $166^a$(R -3 2/m) | a=3.56141 Å<br>b=3.56141 Å<br>c=21.99416 Å<br>$\alpha=\beta=90°,\gamma=120°$ | Au 0.00000 0.00000 0.00000<br>Se 0.00000 0.00000 -0.11227<br>Co 0.00000 0.00000 0.50000 |
| $AuCrSe_2$ | $166^a$(R -3 2/m) | a=3.74847 Å<br>b=3.74847 Å<br>c=22.57021 Å<br>$\alpha=\beta=90°,\gamma=120°$ | Au 0.00000 0.00000 0.00000<br>Se 0.00000 0.00000 -0.10836<br>Cr 0.00000 0.00000 0.50000 |
| $AuMnSe_2$ | $166^a$(R -3 2/m) | a=3.79353 Å<br>b=3.79353 Å<br>c=22.78084 Å<br>$\alpha=\beta=90°,\gamma=120°$ | Au 0.00000 0.00000 0.00000<br>Se 0.00000 0.00000 -0.10703<br>Mn 0.00000 0.00000 0.50000 |
| $AuRhSe_2$ | $166^a$(R -3 2/m) | a=3.76914 Å<br>b=3.76914 Å<br>c=22.11265 Å<br>$\alpha=\beta=90°,\gamma=120°$ | Au 0.00000 0.00000 0.00000<br>Rh 0.00000 0.00000 0.50000<br>Se 0.00000 0.00000 -0.11075 |
| $HgHfSe_2$ | $166^b$(R -3 2/m) | a=3.75047 Å<br>b=3.75047 Å<br>c=29.09357 Å<br>$\alpha=\beta=90°,\gamma=120°$ | Hg 0.00000 0.00000 0.00000<br>Hf 0.00000 0.00000 0.50000<br>Se 0.00000 0.00000 -0.22118 |
| $HgMnSe_2$ | $166^b$(R -3 2/m) | a=3.48295 Å<br>b=3.48295 Å<br>c=28.09625 Å<br>$\alpha=\beta=90°,\gamma=120°$ | Hg 0.00000 0.00000 0.50000<br>Se 0.00000 0.00000 0.28205<br>Mn 0.00000 0.00000 0.00000 |
| $HgNbSe_2$ | 38(A m m 2) | a=9.60790 Å<br>b=3.47683 Å<br>c=6.01235 Å<br>$\alpha=\beta=90°,\gamma=90°$ | Hg 0.50000 0.00000 0.01408<br>Nb 0.00000 0.00000 0.33580<br>Se -0.17541 0.00000 -0.33075 |
| $HgPtSe_2$ | $166^b$(R -3 2/m) | a=3.74663 Å<br>b=3.74663 Å<br>c=26.63660 Å<br>$\alpha=\beta=90°,\gamma=120°$ | Hg 0.00000 0.00000 0.50000<br>Pt 0.00000 0.00000 0.00000<br>Se 0.00000 0.00000 0.28394 |

Table S9: Structual parameters of the new stable delafossite and closely related phases with composition $ABSe_2$. Continuation of Table S8.

| | | | |
|---|---|---|---|
| $HgRhSe_2$ | $166^a$(R -3 2/m) | a=3.80643 Å<br>b=3.80643 Å<br>c=23.93630 Å<br>$\alpha$=$\beta$=90°,$\gamma$=120° | Hg 0.00000 0.00000 0.00000<br>Rh 0.00000 0.00000 0.50000<br>Se 0.00000 0.00000 -0.11563 |
| $HgTiSe_2$ | 1 (P 1) | a=3.52743 Å<br>b=10.10198 Å<br>c=6.11632 Å<br>$\alpha$=102.59452°<br>$\beta$=90°<br>$\gamma$=99.61212° | Hg 0.48447 0.00794 -0.02072<br>Hg 0.47672 -0.00430 0.47278<br>Se -0.23259 -0.33594 0.16527<br>Se 0.11387 0.34262 0.21573<br>Se 0.26668 -0.33700 -0.33558<br>Se -0.38583 0.34373 -0.28350<br>Ti 0.19058 -0.49659 -0.05892<br>Ti -0.30968 -0.49673 0.44005 |
| $HgVSe_2$ | 6 (P 1 m 1) | a=3.33180 Å<br>b=9.78877 Å<br>c=3.32996 Å<br>$\alpha$=$\gamma$=90°, $\beta$=90° | Hg -0.16355 0.00000 0.25366<br>Se 0.44473 0.33673 -0.11923<br>V 0.11095 0.50000 -0.45296 |
| $HgZrSe_2$ | $166^b$(R -3 2/m) | a=3.78211 Å<br>b=3.78211 Å<br>c=28.37739 Å<br>$\alpha$=$\beta$=90°,$\gamma$=120° | Hg 0.00000 0.00000 0.00000<br>Zr 0.00000 0.00000 0.50000<br>Se 0.00000 0.00000 -0.22324 |
| $InZrSe_2$ | 164 (P -3 2/m 1) | a=b=3.74384 Å<br>c=8.43595 Å<br>$\alpha$=$\beta$=90°,$\gamma$=120° | In 0.00000 0.00000 0.00000<br>Zr 0.00000 0.00000 0.50000<br>Se 0.33333 0.66667 -0.30261 |
| $TlHfSe_2$ | $164^b$(P -3 2/m 1) | a=3.72705 Å<br>b=3.72705 Å<br>c=8.77741 Å<br>$\alpha$=$\beta$=90°,$\gamma$=120° | Tl 0.00000 0.00000 0.50000<br>Hf 0.00000 0.00000 0.00000<br>Se 0.33333 0.66667 0.18439 |
| $TlMnSe_2$ | $166^b$(R -3 2/m) | a=3.83420 Å<br>b=3.83420 Å<br>c=22.61324 Å<br>$\alpha$=$\beta$=90°,$\gamma$=120° | Tl 0.00000 0.00000 0.50000<br>Se 0.00000 0.00000 -0.27216<br>Mn 0.00000 0.00000 0.00000 |
| $TlRhSe_2$ | 164 (P -3 2/m 1) | a=3.62640 Å<br>b=3.62640 Å<br>c=8.41867 Å<br>$\alpha$=$\beta$=90°,$\gamma$=120° | Tl 0.00000 0.00000 0.00000<br>Se 0.33333 0.66667 0.31829<br>Rh 0.00000 0.00000 0.50000 |
| $TlTiSe_2$ | 164 (P -3 2/m 1) | a=b=3.62640 Å<br>c=8.41867 Å<br>$\alpha$=$\beta$=90°,$\gamma$=120° | Tl 0.00000 0.00000 0.00000<br>Se 0.33333 0.66667 0.31829<br>Ti 0.00000 0.00000 0.50000 |
| $TlVSe2_2$ | 187 (P -6 m 2) | a=b=3.42683 Å<br>c=8.66380 Å<br>$\alpha$=$\beta$=90°,$\gamma$=120° | Tl 0.33333 0.66667 0.00000<br>Se 0.00000 0.00000 -0.32134<br>V 0.33333 0.66667 0.50000 |
| $TlZrSe_2$ | 164 (P -3 2/m 1) | a=b=3.78264 Å<br>c=8.47906 Å<br>$\alpha$=$\beta$=90°,$\gamma$=120° | Tl 0.00000 0.00000 0.50000<br>Zr 0.00000 0.00000 0.00000<br>Se 0.33333 0.66667 -0.19389 |

Table S10: Structual parameters of the new stable delafossite and closely related phases with composition $ABSe_2$. Continuation of Table S9.

| | | | |
|---|---|---|---|
| $SbMnSe_2$ | 11 (P 1 21/m 1) | a=8.59123 Å<br>b=3.92579 Å<br>c=6.32267 Å<br>$\alpha$=$\gamma$=90°<br>$\beta$=75.92688° | Sb -0.02917 0.25000 -0.15519<br>Se 0.32357 0.25000 0.47176<br>Se -0.34643 0.25000 0.03697<br>Mn -0.49235 0.25000 -0.27482 |
| $BiCrSe_2$ | 166[a](R -3 2/m) | a=3.65977 Å<br>b=3.65977 Å<br>c=25.89903 Å<br>$\alpha$=$\beta$=90°,$\gamma$=120° | Bi 0.00000 0.00000 0.00000<br>Se 0.00000 0.00000 -0.11356<br>Cr 0.00000 0.00000 0.50000 |
| $BiMnSe_2$ | 166[a](R -3 2/m) | a=3.63018 Å<br>b=3.63018 Å<br>c=26.29310 Å<br>$\alpha$=$\beta$=90°,$\gamma$=120° | Bi 0.00000 0.00000 0.50000<br>Se 0.00000 0.00000 -0.38732<br>Mn 0.00000 0.00000 0.00000 |
| $BrNiSe_2$ | 166[a](R -3 2/m) | a=3.70682 Å<br>b=3.70682 Å<br>c=22.84750 Å<br>$\alpha$=$\beta$=90°,$\gamma$=120° | Br 0.00000 0.00000 0.00000<br>Se 0.00000 0.00000 -0.11562<br>Ni 0.00000 0.00000 0.50000 |
| $INiSe_2$ | 166[a](R -3 2/m) | a=3.75533 Å<br>b=3.75533 Å<br>c=23.93427 Å<br>$\alpha$=$\beta$=90°,$\gamma$=120° | I 0.00000 0.00000 0.00000<br>Se 0.00000 0.00000 -0.11809<br>Ni 0.00000 0.00000 0.50000 |

Table S11: Structual parameters of the new stable delafossite and closely related phases with composition $ABTe_2$.

| | | | |
|---|---|---|---|
| $LiYTe_2$ | 164 (P -3 2/m 1) | a=b=4.30381 Å<br>c=7.15747 Å<br>$\alpha=\beta=90°,\gamma=120°$ | Te 0.33333 0.66667 -0.25725<br>Y 0.00000 0.00000 0.00000<br>Li 0.00000 0.00000 0.50000 |
| $NaYTe_2$ | $166^b$(R -3 2/m) | a=4.43028 Å<br>b=4.43028 Å<br>c=22.70594 Å<br>$\alpha=\beta=90°,\gamma=120°$ | Te 0.00000 0.00000 0.24412<br>Y 0.00000 0.00000 0.50000<br>Na 0.00000 0.00000 0.00000 |
| $RbLaTe_2$ | $166^b$(R -3 2/m) | a=4.74251 Å<br>b=4.74251 Å<br>c=25.61822 Å<br>$\alpha=\beta=90°,\gamma=120°$ | La 0.00000 0.00000 0.50000<br>Te 0.00000 0.00000 -0.23545<br>Rb 0.00000 0.00000 0.00000 |
| $CsBiTe_2$ | $166^b$(R -3 2/m) | a=4.65619 Å<br>b=4.65619 Å<br>c=26.88465 Å<br>$\alpha=\beta=90°,\gamma=120°$ | Bi 0.00000 0.00000 0.00000<br>Cs 0.00000 0.00000 0.50000<br>Te 0.00000 0.00000 0.26897 |
| $CsHfTe_2$ | $166^b$(R -3 2/m) | a=4.20620 Å<br>b=4.20620 Å<br>c=27.05162 Å<br>$\alpha=\beta=90°,\gamma=120°$ | Hf 0.00000 0.00000 0.00000<br>Cs 0.00000 0.00000 0.50000<br>Te 0.00000 0.00000 0.27177 |
| $CsScTe_2$ | $166^b$(R -3 2/m) | a=4.37131 Å<br>b=4.37131 Å<br>c=26.50526 Å<br>$\alpha=\beta=90°,\gamma=120°$ | Cs 0.00000 0.00000 0.00000<br>Te 0.00000 0.00000 0.22681<br>Sc 0.00000 0.00000 0.50000 |
| $CsYTe_2$ | $166^b$(R -3 2/m) | a=4.55740 Å<br>b=4.55740 Å<br>c=26.74329 Å<br>$\alpha=\beta=90°,\gamma=120°$ | Cs 0.00000 0.00000 0.50000<br>Te 0.00000 0.00000 0.26999<br>Y 0.00000 0.00000 0.00000 |
| $BaCaTe_2$ | $166^b$(R -3 2/m) | a=4.76440 Å<br>b=4.76440 Å<br>c=23.39713 Å<br>$\alpha=\beta=90°,\gamma=120°$ | Ba 0.00000 0.00000 0.00000<br>Te 0.00000 0.00000 -0.24129<br>Ca 0.00000 0.00000 0.50000 |
| $AgMnTe_2$ | $166^b$(R -3 2/m) | a=4.03881 Å<br>b=4.03881 Å<br>c=20.81605 Å<br>$\alpha=\beta=90°,\gamma=120°$ | Te 0.00000 0.00000 0.24288<br>Ag 0.00000 0.00000 0.00000<br>Mn 0.00000 0.00000 0.50000 |
| $HgHfTe_2$ | $166^b$(R -3 2/m) | a=3.95145 Å<br>b=3.95145 Å<br>c=29.71098 Å<br>$\alpha=\beta=90°,\gamma=120°$ | Hg 0.00000 0.00000 0.00000<br>Hf 0.00000 0.00000 0.50000<br>Te 0.00000 0.00000 0.22664 |
| $HgTiTe_2$ | $166^b$(R -3 2/m) | a=3.73742 Å<br>b=3.73742 Å<br>c=29.77145 Å<br>$\alpha=\beta=90°,\gamma=120°$ | Hg 0.00000 0.00000 0.50000<br>Te 0.00000 0.00000 0.27456<br>Ti 0.00000 0.00000 0.00000 |

Table S12: Structual parameters of the new stable delafossite and closely related phases with composition $ABTe_2$. Continuation of Table S11.

| | | | |
|---|---|---|---|
| $HgVTe_2$ | 11 (P 1 21/m 1) | a=6.51190 Å<br>b=3.56739 Å<br>c=10.08520 Å<br>$\alpha$=$\gamma$=90°<br>$\beta$=103.2329° | Hg -0.27846 0.25000 -0.01198<br>Te -0.47984 0.25000 0.34788<br>Te -0.01042 0.25000 -0.30972<br>V 0.20860 0.25000 0.49595 |
| $HgZrTe_2$ | $166^b$(R -3 2/m) | a=3.96118 Å<br>b=3.96118 Å<br>c=28.83456 Å<br>$\alpha$=$\beta$=90°,$\gamma$=120° | Hg 0.00000 0.00000 0.50000<br>Te 0.00000 0.00000 0.27047<br>Zr 0.00000 0.00000 0.00000 |
| $InYTe_2$ | $166^n$(R -3 2/m) | a=4.41664 Å<br>b=4.41664 Å<br>c=24.30902 Å<br>$\alpha$=$\beta$=90°,$\gamma$=120° | Te 0.00000 0.00000 0.26013<br>In 0.00000 0.00000 0.50000<br>Y 0.00000 0.00000 0.00000 |
| $TlHfTe_2$ | 164 (P -3 2/m 1) | a=b=3.98106 Å<br>c=8.97218 Å<br>$\alpha$=$\beta$=90°,$\gamma$=120° | Tl 0.00000 0.00000 0.00000<br>Hf 0.00000 0.00000 0.50000<br>Te 0.33333 0.66667 0.30201 |
| $TlMnTe_2$ | 164 (P -3 2/m 1) | a=b=4.09771 Å<br>c=8.03708 Å<br>$\alpha$=$\beta$=90°,$\gamma$=120° | Tl 0.00000 0.00000 0.00000<br>Te 0.33333 0.66667 0.31205<br>Mn 0.00000 0.00000 0.50000 |
| $TlTiTe_2$ | 164 (P -3 2/m 1) | a=b=3.87987 Å<br>c=8.81594 Å<br>$\alpha$=$\beta$=90°,$\gamma$=120° | Tl 0.00000 0.00000 0.50000<br>Te 0.33333 0.66667 -0.19086<br>Ti 0.00000 0.00000 0.00000 |
| $TlYTe_2$ | $166^b$(R -3 2/m) | a=4.45463 Å<br>b=4.45463 Å<br>c=24.55466 Å<br>$\alpha$=$\beta$=90°,$\gamma$=120° | Tl 0.00000 0.00000 0.50000<br>Te 0.00000 0.00000 0.26210<br>Y 0.00000 0.00000 0.00000 |
| $TlZrTe_2$ | 164 (P -3 2/m 1) | a=b=4.01882 Å<br>c= 8.87565 Å<br>$\alpha$=$\beta$=90°,$\gamma$=120° | Tl 0.00000 0.00000 0.00000<br>Te 0.33333 0.66667 -0.29706<br>Zr 0.00000 0.00000 0.50000 |
| $BrCoTe_2$ | $166^a$(R -3 2/m) | a=3.92958 Å<br>b=3.92958 Å<br>c=24.25180 Å<br>$\alpha$=$\beta$=90°,$\gamma$=120° | Te 0.00000 0.00000 -0.11673<br>Br 0.00000 0.00000 0.00000<br>Co 0.00000 0.00000 0.50000 |
| $BrIrTe_2$ | 160 (R 3 m)<br>(hexagonal axes) | a=b=4.11707 Å<br>c=25.57033 Å<br>$\alpha$=$\beta$=90°,$\gamma$=120° | Ir 0.00000 0.00000 -0.33956<br>Te 0.00000 0.00000 0.27253<br>Te 0.00000 0.00000 0.03659<br>Br 0.00000 0.00000 0.13794 |
| $BrNiTe_2$ | $166^b$(R -3 2/m) | a=3.89925 Å<br>b=3.89925 Å<br>c=22.24186 Å<br>$\alpha$=$\beta$=90°,$\gamma$=120° | Te 0.00000 0.00000 -0.27675<br>Br 0.00000 0.00000 0.50000<br>Ni 0.00000 0.00000 0.00000 |

Table S13: Structual parameters of the new stable delafossite and closely related phases with composition $ABTe_2$. Continuation of Table S11.

| | | | |
|---|---|---|---|
| $BrPdTe_2$ | 12 (A 1 2/m 1) | a=9.3950 Å,b=4.1186 Å<br>c=15.46901 Å<br>$\alpha=\gamma=90°$<br>$\beta=158.55°$ | Te 0.21339 0.00000 0.01870<br>Pd 0.00000 0.50000 0.00000<br>Br 0.50000 0.00000 0.50000 |
| $BrPtTe_2$ | $166^b$(R -3 2/m) | a=4.09656 Å<br>b=4.09656 Å<br>c=22.19780 Å<br>$\alpha=\beta=90°,\gamma=120°$ | Pt 0.00000 0.00000 0.50000<br>Te 0.00000 0.00000 0.22624<br>Br 0.00000 0.00000 0.00000 |
| $BrRhTe_2$ | $166^a$(R -3 2/m) | a=4.13050 Å<br>b=4.13050 Å<br>c=24.26323 Å<br>$\alpha=\beta=90°,\gamma=120°$ | Te 0.00000 0.00000 -0.11598<br>Rh 0.00000 0.00000 0.50000<br>Br 0.00000 0.00000 0.00000 |
| $INiTe_2$ | 13 (P 1 2/n 1) | a=8.9478 Å,b=3.9454 Å<br>c=8.14783 Å<br>$\alpha=\gamma=90°$<br>$\beta=133.06°$ | I 0.75000 -0.24968 0.75000<br>Te 0.02430 0.25030 -0.31515<br>Ni 0.75000 0.24964 0.25000 |
| $IPdTe_2$ | 12 (A 1 2/m 1) | a=9.61944 Å,b=4.15255 Å<br>c=7.20406 Å<br>$\alpha=\gamma=90°$<br>$\beta=126.20°$ | I 0.50000 0.50000 0.50000<br>Te -0.16809 0.00000 -0.47290<br>Pd 0.00000 0.00000 0.00000 |
| $IPtTe_2$ | $166^b$(R -3 2/m) | a=4.11626 Å<br>b=4.11626 Å<br>c=23.44422 Å<br>$\alpha=\beta=90°,\gamma=120°$ | Pt 0.00000 0.00000 0.00000<br>I 0.00000 0.00000 0.50000<br>Te 0.00000 0.00000 0.27680 |
| $IRhTe_2$ | $166^a$(R -3 2/m) | a=4.14203 Å<br>b=4.14203 Å<br>c=25.37537 Å<br>$\alpha=\beta=90°,\gamma=120°$ | I 0.00000 0.00000 0.00000<br>Te 0.00000 0.00000 -0.11790<br>Rh 0.00000 0.00000 0.50000 |
